# Search for bottom squark pair production in proton–proton collisions at $\sqrt{s}=13$ TeV with the ATLAS detector

**DOI:** 10.1140/epjc/s10052-016-4382-4

**Published:** 2016-10-06

**Authors:** M. Aaboud, G. Aad, B. Abbott, J. Abdallah, O. Abdinov, B. Abeloos, R. Aben, O. S. AbouZeid, N. L. Abraham, H. Abramowicz, H. Abreu, R. Abreu, Y. Abulaiti, B. S. Acharya, L. Adamczyk, D. L. Adams, J. Adelman, S. Adomeit, T. Adye, A. A. Affolder, T. Agatonovic-Jovin, J. Agricola, J. A. Aguilar-Saavedra, S. P. Ahlen, F. Ahmadov, G. Aielli, H. Akerstedt, T. P. A. Åkesson, A. V. Akimov, G. L. Alberghi, J. Albert, S. Albrand, M. J. Alconada Verzini, M. Aleksa, I. N. Aleksandrov, C. Alexa, G. Alexander, T. Alexopoulos, M. Alhroob, B. Ali, M. Aliev, G. Alimonti, J. Alison, S. P. Alkire, B. M. M. Allbrooke, B. W. Allen, P. P. Allport, A. Aloisio, A. Alonso, F. Alonso, C. Alpigiani, M. Alstaty, B. Alvarez Gonzalez, D. Álvarez Piqueras, M. G. Alviggi, B. T. Amadio, K. Amako, Y. Amaral Coutinho, C. Amelung, D. Amidei, S. P. Amor Dos Santos, A. Amorim, S. Amoroso, G. Amundsen, C. Anastopoulos, L. S. Ancu, N. Andari, T. Andeen, C. F. Anders, G. Anders, J. K. Anders, K. J. Anderson, A. Andreazza, V. Andrei, S. Angelidakis, I. Angelozzi, P. Anger, A. Angerami, F. Anghinolfi, A. V. Anisenkov, N. Anjos, A. Annovi, C. Antel, M. Antonelli, A. Antonov, F. Anulli, M. Aoki, L. Aperio Bella, G. Arabidze, Y. Arai, J. P. Araque, A. T. H. Arce, F. A. Arduh, J.-F. Arguin, S. Argyropoulos, M. Arik, A. J. Armbruster, L. J. Armitage, O. Arnaez, H. Arnold, M. Arratia, O. Arslan, A. Artamonov, G. Artoni, S. Artz, S. Asai, N. Asbah, A. Ashkenazi, B. Åsman, L. Asquith, K. Assamagan, R. Astalos, M. Atkinson, N. B. Atlay, K. Augsten, G. Avolio, B. Axen, M. K. Ayoub, G. Azuelos, M. A. Baak, A. E. Baas, M. J. Baca, H. Bachacou, K. Bachas, M. Backes, M. Backhaus, P. Bagiacchi, P. Bagnaia, Y. Bai, J. T. Baines, O. K. Baker, E. M. Baldin, P. Balek, T. Balestri, F. Balli, W. K. Balunas, E. Banas, Sw. Banerjee, A. A. E. Bannoura, L. Barak, E. L. Barberio, D. Barberis, M. Barbero, T. Barillari, M.-S. Barisits, T. Barklow, N. Barlow, S. L. Barnes, B. M. Barnett, R. M. Barnett, Z. Barnovska, A. Baroncelli, G. Barone, A. J. Barr, L. Barranco Navarro, F. Barreiro, J. Barreiro Guimarães da Costa, R. Bartoldus, A. E. Barton, P. Bartos, A. Basalaev, A. Bassalat, R. L. Bates, S. J. Batista, J. R. Batley, M. Battaglia, M. Bauce, F. Bauer, H. S. Bawa, J. B. Beacham, M. D. Beattie, T. Beau, P. H. Beauchemin, P. Bechtle, H. P. Beck, K. Becker, M. Becker, M. Beckingham, C. Becot, A. J. Beddall, A. Beddall, V. A. Bednyakov, M. Bedognetti, C. P. Bee, L. J. Beemster, T. A. Beermann, M. Begel, J. K. Behr, C. Belanger-Champagne, A. S. Bell, G. Bella, L. Bellagamba, A. Bellerive, M. Bellomo, K. Belotskiy, O. Beltramello, N. L. Belyaev, O. Benary, D. Benchekroun, M. Bender, K. Bendtz, N. Benekos, Y. Benhammou, E. Benhar Noccioli, J. Benitez, D. P. Benjamin, J. R. Bensinger, S. Bentvelsen, L. Beresford, M. Beretta, D. Berge, E. Bergeaas Kuutmann, N. Berger, J. Beringer, S. Berlendis, N. R. Bernard, C. Bernius, F. U. Bernlochner, T. Berry, P. Berta, C. Bertella, G. Bertoli, F. Bertolucci, I. A. Bertram, C. Bertsche, D. Bertsche, G. J. Besjes, O. Bessidskaia Bylund, M. Bessner, N. Besson, C. Betancourt, A. Bethani, S. Bethke, A. J. Bevan, R. M. Bianchi, L. Bianchini, M. Bianco, O. Biebel, D. Biedermann, R. Bielski, N. V. Biesuz, M. Biglietti, J. Bilbao De Mendizabal, T. R. V. Billoud, H. Bilokon, M. Bindi, S. Binet, A. Bingul, C. Bini, S. Biondi, T. Bisanz, D. M. Bjergaard, C. W. Black, J. E. Black, K. M. Black, D. Blackburn, R. E. Blair, J.-B. Blanchard, T. Blazek, I. Bloch, C. Blocker, W. Blum, U. Blumenschein, S. Blunier, G. J. Bobbink, V. S. Bobrovnikov, S. S. Bocchetta, A. Bocci, C. Bock, M. Boehler, D. Boerner, J. A. Bogaerts, D. Bogavac, A. G. Bogdanchikov, C. Bohm, V. Boisvert, P. Bokan, T. Bold, A. S. Boldyrev, M. Bomben, M. Bona, M. Boonekamp, A. Borisov, G. Borissov, J. Bortfeldt, D. Bortoletto, V. Bortolotto, K. Bos, D. Boscherini, M. Bosman, J. D. Bossio Sola, J. Boudreau, J. Bouffard, E. V. Bouhova-Thacker, D. Boumediene, C. Bourdarios, S. K. Boutle, A. Boveia, J. Boyd, I. R. Boyko, J. Bracinik, A. Brandt, G. Brandt, O. Brandt, U. Bratzler, B. Brau, J. E. Brau, H. M. Braun, W. D. Breaden Madden, K. Brendlinger, A. J. Brennan, L. Brenner, R. Brenner, S. Bressler, T. M. Bristow, D. Britton, D. Britzger, F. M. Brochu, I. Brock, R. Brock, G. Brooijmans, T. Brooks, W. K. Brooks, J. Brosamer, E. Brost, J. H Broughton, P. A. Bruckman de Renstrom, D. Bruncko, R. Bruneliere, A. Bruni, G. Bruni, L. S. Bruni, B. H. Brunt, M. Bruschi, N. Bruscino, P. Bryant, L. Bryngemark, T. Buanes, Q. Buat, P. Buchholz, A. G. Buckley, I. A. Budagov, F. Buehrer, M. K. Bugge, O. Bulekov, D. Bullock, H. Burckhart, S. Burdin, C. D. Burgard, B. Burghgrave, K. Burka, S. Burke, I. Burmeister, J. T. P. Burr, E. Busato, D. Büscher, V. Büscher, P. Bussey, J. M. Butler, C. M. Buttar, J. M. Butterworth, P. Butti, W. Buttinger, A. Buzatu, A. R. Buzykaev, S. Cabrera Urbán, D. Caforio, V. M. Cairo, O. Cakir, N. Calace, P. Calafiura, A. Calandri, G. Calderini, P. Calfayan, G. Callea, L. P. Caloba, S. Calvente Lopez, D. Calvet, S. Calvet, T. P. Calvet, R. Camacho Toro, S. Camarda, P. Camarri, D. Cameron, R. Caminal Armadans, C. Camincher, S. Campana, M. Campanelli, A. Camplani, A. Campoverde, V. Canale, A. Canepa, M. Cano Bret, J. Cantero, R. Cantrill, T. Cao, M. D. M. Capeans Garrido, I. Caprini, M. Caprini, M. Capua, R. Caputo, R. M. Carbone, R. Cardarelli, F. Cardillo, I. Carli, T. Carli, G. Carlino, L. Carminati, S. Caron, E. Carquin, G. D. Carrillo-Montoya, J. R. Carter, J. Carvalho, D. Casadei, M. P. Casado, M. Casolino, D. W. Casper, E. Castaneda-Miranda, R. Castelijn, A. Castelli, V. Castillo Gimenez, N. F. Castro, A. Catinaccio, J. R. Catmore, A. Cattai, J. Caudron, V. Cavaliere, E. Cavallaro, D. Cavalli, M. Cavalli-Sforza, V. Cavasinni, F. Ceradini, L. Cerda Alberich, B. C. Cerio, A. S. Cerqueira, A. Cerri, L. Cerrito, F. Cerutti, M. Cerv, A. Cervelli, S. A. Cetin, A. Chafaq, D. Chakraborty, S. K. Chan, Y. L. Chan, P. Chang, J. D. Chapman, D. G. Charlton, A. Chatterjee, C. C. Chau, C. A. Chavez Barajas, S. Che, S. Cheatham, A. Chegwidden, S. Chekanov, S. V. Chekulaev, G. A. Chelkov, M. A. Chelstowska, C. Chen, H. Chen, K. Chen, S. Chen, S. Chen, X. Chen, Y. Chen, H. C. Cheng, H. J Cheng, Y. Cheng, A. Cheplakov, E. Cheremushkina, R. Cherkaoui El Moursli, V. Chernyatin, E. Cheu, L. Chevalier, V. Chiarella, G. Chiarelli, G. Chiodini, A. S. Chisholm, A. Chitan, M. V. Chizhov, K. Choi, A. R. Chomont, S. Chouridou, B. K. B. Chow, V. Christodoulou, D. Chromek-Burckhart, J. Chudoba, A. J. Chuinard, J. J. Chwastowski, L. Chytka, G. Ciapetti, A. K. Ciftci, D. Cinca, V. Cindro, I. A. Cioara, C. Ciocca, A. Ciocio, F. Cirotto, Z. H. Citron, M. Citterio, M. Ciubancan, A. Clark, B. L. Clark, M. R. Clark, P. J. Clark, R. N. Clarke, C. Clement, Y. Coadou, M. Cobal, A. Coccaro, J. Cochran, L. Colasurdo, B. Cole, A. P. Colijn, J. Collot, T. Colombo, G. Compostella, P. Conde Muiño, E. Coniavitis, S. H. Connell, I. A. Connelly, V. Consorti, S. Constantinescu, G. Conti, F. Conventi, M. Cooke, B. D. Cooper, A. M. Cooper-Sarkar, K. J. R. Cormier, T. Cornelissen, M. Corradi, F. Corriveau, A. Corso-Radu, A. Cortes-Gonzalez, G. Cortiana, G. Costa, M. J. Costa, D. Costanzo, G. Cottin, G. Cowan, B. E. Cox, K. Cranmer, S. J. Crawley, G. Cree, S. Crépé-Renaudin, F. Crescioli, W. A. Cribbs, M. Crispin Ortuzar, M. Cristinziani, V. Croft, G. Crosetti, A. Cueto, T. Cuhadar Donszelmann, J. Cummings, M. Curatolo, J. Cúth, H. Czirr, P. Czodrowski, G. D’amen, S. D’Auria, M. D’Onofrio, M. J. Da Cunha Sargedas De Sousa, C. Da Via, W. Dabrowski, T. Dado, T. Dai, O. Dale, F. Dallaire, C. Dallapiccola, M. Dam, J. R. Dandoy, N. P. Dang, A. C. Daniells, N. S. Dann, M. Danninger, M. Dano Hoffmann, V. Dao, G. Darbo, S. Darmora, J. Dassoulas, A. Dattagupta, W. Davey, C. David, T. Davidek, M. Davies, P. Davison, E. Dawe, I. Dawson, R. K. Daya-Ishmukhametova, K. De, R. de Asmundis, A. De Benedetti, S. De Castro, S. De Cecco, N. De Groot, P. de Jong, H. De la Torre, F. De Lorenzi, A. De Maria, D. De Pedis, A. De Salvo, U. De Sanctis, A. De Santo, J. B. De Vivie De Regie, W. J. Dearnaley, R. Debbe, C. Debenedetti, D. V. Dedovich, N. Dehghanian, I. Deigaard, M. Del Gaudio, J. Del Peso, T. Del Prete, D. Delgove, F. Deliot, C. M. Delitzsch, A. Dell’Acqua, L. Dell’Asta, M. Dell’Orso, M. Della Pietra, D. della Volpe, M. Delmastro, P. A. Delsart, D. A. DeMarco, S. Demers, M. Demichev, A. Demilly, S. P. Denisov, D. Denysiuk, D. Derendarz, J. E. Derkaoui, F. Derue, P. Dervan, K. Desch, C. Deterre, K. Dette, P. O. Deviveiros, A. Dewhurst, S. Dhaliwal, A. Di Ciaccio, L. Di Ciaccio, W. K. Di Clemente, C. Di Donato, A. Di Girolamo, B. Di Girolamo, B. Di Micco, R. Di Nardo, A. Di Simone, R. Di Sipio, D. Di Valentino, C. Diaconu, M. Diamond, F. A. Dias, M. A. Diaz, E. B. Diehl, J. Dietrich, S. Diglio, A. Dimitrievska, J. Dingfelder, P. Dita, S. Dita, F. Dittus, F. Djama, T. Djobava, J. I. Djuvsland, M. A. B. do Vale, D. Dobos, M. Dobre, C. Doglioni, J. Dolejsi, Z. Dolezal, M. Donadelli, S. Donati, P. Dondero, J. Donini, J. Dopke, A. Doria, M. T. Dova, A. T. Doyle, E. Drechsler, M. Dris, Y. Du, J. Duarte-Campderros, E. Duchovni, G. Duckeck, O. A. Ducu, D. Duda, A. Dudarev, A. Chr. Dudder, E. M. Duffield, L. Duflot, M. Dührssen, M. Dumancic, M. Dunford, H. Duran Yildiz, M. Düren, A. Durglishvili, D. Duschinger, B. Dutta, M. Dyndal, C. Eckardt, K. M. Ecker, R. C. Edgar, N. C. Edwards, T. Eifert, G. Eigen, K. Einsweiler, T. Ekelof, M. El Kacimi, V. Ellajosyula, M. Ellert, S. Elles, F. Ellinghaus, A. A. Elliot, N. Ellis, J. Elmsheuser, M. Elsing, D. Emeliyanov, Y. Enari, O. C. Endner, J. S. Ennis, J. Erdmann, A. Ereditato, G. Ernis, J. Ernst, M. Ernst, S. Errede, E. Ertel, M. Escalier, H. Esch, C. Escobar, B. Esposito, A. I. Etienvre, E. Etzion, H. Evans, A. Ezhilov, F. Fabbri, L. Fabbri, G. Facini, R. M. Fakhrutdinov, S. Falciano, R. J. Falla, J. Faltova, Y. Fang, M. Fanti, A. Farbin, A. Farilla, C. Farina, E. M. Farina, T. Farooque, S. Farrell, S. M. Farrington, P. Farthouat, F. Fassi, P. Fassnacht, D. Fassouliotis, M. Faucci Giannelli, A. Favareto, W. J. Fawcett, L. Fayard, O. L. Fedin, W. Fedorko, S. Feigl, L. Feligioni, C. Feng, E. J. Feng, H. Feng, A. B. Fenyuk, L. Feremenga, P. Fernandez Martinez, S. Fernandez Perez, J. Ferrando, A. Ferrari, P. Ferrari, R. Ferrari, D. E. Ferreira de Lima, A. Ferrer, D. Ferrere, C. Ferretti, A. Ferretto Parodi, F. Fiedler, A. Filipčič, M. Filipuzzi, F. Filthaut, M. Fincke-Keeler, K. D. Finelli, M. C. N. Fiolhais, L. Fiorini, A. Firan, A. Fischer, C. Fischer, J. Fischer, W. C. Fisher, N. Flaschel, I. Fleck, P. Fleischmann, G. T. Fletcher, R. R. M. Fletcher, T. Flick, A. Floderus, L. R. Flores Castillo, M. J. Flowerdew, G. T. Forcolin, A. Formica, A. Forti, A. G. Foster, D. Fournier, H. Fox, S. Fracchia, P. Francavilla, M. Franchini, D. Francis, L. Franconi, M. Franklin, M. Frate, M. Fraternali, D. Freeborn, S. M. Fressard-Batraneanu, F. Friedrich, D. Froidevaux, J. A. Frost, C. Fukunaga, E. Fullana Torregrosa, T. Fusayasu, J. Fuster, C. Gabaldon, O. Gabizon, A. Gabrielli, A. Gabrielli, G. P. Gach, S. Gadatsch, S. Gadomski, G. Gagliardi, L. G. Gagnon, P. Gagnon, C. Galea, B. Galhardo, E. J. Gallas, B. J. Gallop, P. Gallus, G. Galster, K. K. Gan, J. Gao, Y. Gao, Y. S. Gao, F. M. Garay Walls, C. García, J. E. García Navarro, M. Garcia-Sciveres, R. W. Gardner, N. Garelli, V. Garonne, A. Gascon Bravo, K. Gasnikova, C. Gatti, A. Gaudiello, G. Gaudio, L. Gauthier, I. L. Gavrilenko, C. Gay, G. Gaycken, E. N. Gazis, Z. Gecse, C. N. P. Gee, Ch. Geich-Gimbel, M. Geisen, M. P. Geisler, C. Gemme, M. H. Genest, C. Geng, S. Gentile, C. Gentsos, S. George, D. Gerbaudo, A. Gershon, S. Ghasemi, H. Ghazlane, M. Ghneimat, B. Giacobbe, S. Giagu, P. Giannetti, B. Gibbard, S. M. Gibson, M. Gignac, M. Gilchriese, T. P. S. Gillam, D. Gillberg, G. Gilles, D. M. Gingrich, N. Giokaris, M. P. Giordani, F. M. Giorgi, F. M. Giorgi, P. F. Giraud, P. Giromini, D. Giugni, F. Giuli, C. Giuliani, M. Giulini, B. K. Gjelsten, S. Gkaitatzis, I. Gkialas, E. L. Gkougkousis, L. K. Gladilin, C. Glasman, J. Glatzer, P. C. F. Glaysher, A. Glazov, M. Goblirsch-Kolb, J. Godlewski, S. Goldfarb, T. Golling, D. Golubkov, A. Gomes, R. Gonçalo, J. Goncalves Pinto Firmino Da Costa, G. Gonella, L. Gonella, A. Gongadze, S. González de la Hoz, G. Gonzalez Parra, S. Gonzalez-Sevilla, L. Goossens, P. A. Gorbounov, H. A. Gordon, I. Gorelov, B. Gorini, E. Gorini, A. Gorišek, E. Gornicki, A. T. Goshaw, C. Gössling, M. I. Gostkin, C. R. Goudet, D. Goujdami, A. G. Goussiou, N. Govender, E. Gozani, L. Graber, I. Grabowska-Bold, P. O. J. Gradin, P. Grafström, J. Gramling, E. Gramstad, S. Grancagnolo, V. Gratchev, P. M. Gravila, H. M. Gray, E. Graziani, Z. D. Greenwood, C. Grefe, K. Gregersen, I. M. Gregor, P. Grenier, K. Grevtsov, J. Griffiths, A. A. Grillo, K. Grimm, S. Grinstein, Ph. Gris, J.-F. Grivaz, S. Groh, J. P. Grohs, E. Gross, J. Grosse-Knetter, G. C. Grossi, Z. J. Grout, L. Guan, W. Guan, J. Guenther, F. Guescini, D. Guest, O. Gueta, E. Guido, T. Guillemin, S. Guindon, U. Gul, C. Gumpert, J. Guo, Y. Guo, R. Gupta, S. Gupta, G. Gustavino, P. Gutierrez, N. G. Gutierrez Ortiz, C. Gutschow, C. Guyot, C. Gwenlan, C. B. Gwilliam, A. Haas, C. Haber, H. K. Hadavand, N. Haddad, A. Hadef, S. Hageböck, Z. Hajduk, H. Hakobyan, M. Haleem, J. Haley, G. Halladjian, G. D. Hallewell, K. Hamacher, P. Hamal, K. Hamano, A. Hamilton, G. N. Hamity, P. G. Hamnett, L. Han, K. Hanagaki, K. Hanawa, M. Hance, B. Haney, S. Hanisch, P. Hanke, R. Hanna, J. B. Hansen, J. D. Hansen, M. C. Hansen, P. H. Hansen, K. Hara, A. S. Hard, T. Harenberg, F. Hariri, S. Harkusha, R. D. Harrington, P. F. Harrison, F. Hartjes, N. M. Hartmann, M. Hasegawa, Y. Hasegawa, A. Hasib, S. Hassani, S. Haug, R. Hauser, L. Hauswald, M. Havranek, C. M. Hawkes, R. J. Hawkings, D. Hayakawa, D. Hayden, C. P. Hays, J. M. Hays, H. S. Hayward, S. J. Haywood, S. J. Head, T. Heck, V. Hedberg, L. Heelan, S. Heim, T. Heim, B. Heinemann, J. J. Heinrich, L. Heinrich, C. Heinz, J. Hejbal, L. Helary, S. Hellman, C. Helsens, J. Henderson, R. C. W. Henderson, Y. Heng, S. Henkelmann, A. M. Henriques Correia, S. Henrot-Versille, G. H. Herbert, V. Herget, Y. Hernández Jiménez, G. Herten, R. Hertenberger, L. Hervas, G. G. Hesketh, N. P. Hessey, J. W. Hetherly, R. Hickling, E. Higón-Rodriguez, E. Hill, J. C. Hill, K. H. Hiller, S. J. Hillier, I. Hinchliffe, E. Hines, R. R. Hinman, M. Hirose, D. Hirschbuehl, J. Hobbs, N. Hod, M. C. Hodgkinson, P. Hodgson, A. Hoecker, M. R. Hoeferkamp, F. Hoenig, D. Hohn, T. R. Holmes, M. Homann, T. M. Hong, B. H. Hooberman, W. H. Hopkins, Y. Horii, A. J. Horton, J.-Y. Hostachy, S. Hou, A. Hoummada, J. Howarth, M. Hrabovsky, I. Hristova, J. Hrivnac, T. Hryn’ova, A. Hrynevich, C. Hsu, P. J. Hsu, S.-C. Hsu, D. Hu, Q. Hu, S. Hu, Y. Huang, Z. Hubacek, F. Hubaut, F. Huegging, T. B. Huffman, E. W. Hughes, G. Hughes, M. Huhtinen, P. Huo, N. Huseynov, J. Huston, J. Huth, G. Iacobucci, G. Iakovidis, I. Ibragimov, L. Iconomidou-Fayard, E. Ideal, Z. Idrissi, P. Iengo, O. Igonkina, T. Iizawa, Y. Ikegami, M. Ikeno, Y. Ilchenko, D. Iliadis, N. Ilic, T. Ince, G. Introzzi, P. Ioannou, M. Iodice, K. Iordanidou, V. Ippolito, N. Ishijima, M. Ishino, M. Ishitsuka, R. Ishmukhametov, C. Issever, S. Istin, F. Ito, J. M. Iturbe Ponce, R. Iuppa, W. Iwanski, H. Iwasaki, J. M. Izen, V. Izzo, S. Jabbar, B. Jackson, P. Jackson, V. Jain, K. B. Jakobi, K. Jakobs, S. Jakobsen, T. Jakoubek, D. O. Jamin, D. K. Jana, E. Jansen, R. Jansky, J. Janssen, M. Janus, G. Jarlskog, N. Javadov, T. Javůrek, F. Jeanneau, L. Jeanty, J. Jejelava, G.-Y. Jeng, D. Jennens, P. Jenni, C. Jeske, S. Jézéquel, H. Ji, J. Jia, H. Jiang, Y. Jiang, S. Jiggins, J. Jimenez Pena, S. Jin, A. Jinaru, O. Jinnouchi, P. Johansson, K. A. Johns, W. J. Johnson, K. Jon-And, G. Jones, R. W. L. Jones, S. Jones, T. J. Jones, J. Jongmanns, P. M. Jorge, J. Jovicevic, X. Ju, A. Juste Rozas, M. K. Köhler, A. Kaczmarska, M. Kado, H. Kagan, M. Kagan, S. J. Kahn, T. Kaji, E. Kajomovitz, C. W. Kalderon, A. Kaluza, S. Kama, A. Kamenshchikov, N. Kanaya, S. Kaneti, L. Kanjir, V. A. Kantserov, J. Kanzaki, B. Kaplan, L. S. Kaplan, A. Kapliy, D. Kar, K. Karakostas, A. Karamaoun, N. Karastathis, M. J. Kareem, E. Karentzos, M. Karnevskiy, S. N. Karpov, Z. M. Karpova, K. Karthik, V. Kartvelishvili, A. N. Karyukhin, K. Kasahara, L. Kashif, R. D. Kass, A. Kastanas, Y. Kataoka, C. Kato, A. Katre, J. Katzy, K. Kawagoe, T. Kawamoto, G. Kawamura, V. F. Kazanin, R. Keeler, R. Kehoe, J. S. Keller, J. J. Kempster, K Kentaro, H. Keoshkerian, O. Kepka, B. P. Kerševan, S. Kersten, R. A. Keyes, M. Khader, F. Khalil-zada, A. Khanov, A. G. Kharlamov, T. J. Khoo, V. Khovanskiy, E. Khramov, J. Khubua, S. Kido, C. R. Kilby, H. Y. Kim, S. H. Kim, Y. K. Kim, N. Kimura, O. M. Kind, B. T. King, M. King, S. B. King, J. Kirk, A. E. Kiryunin, T. Kishimoto, D. Kisielewska, F. Kiss, K. Kiuchi, O. Kivernyk, E. Kladiva, M. H. Klein, M. Klein, U. Klein, K. Kleinknecht, P. Klimek, A. Klimentov, R. Klingenberg, J. A. Klinger, T. Klioutchnikova, E.-E. Kluge, P. Kluit, S. Kluth, J. Knapik, E. Kneringer, E. B. F. G. Knoops, A. Knue, A. Kobayashi, D. Kobayashi, T. Kobayashi, M. Kobel, M. Kocian, P. Kodys, N. M. Koehler, T. Koffas, E. Koffeman, T. Koi, H. Kolanoski, M. Kolb, I. Koletsou, A. A. Komar, Y. Komori, T. Kondo, N. Kondrashova, K. Köneke, A. C. König, T. Kono, R. Konoplich, N. Konstantinidis, R. Kopeliansky, S. Koperny, L. Köpke, A. K. Kopp, K. Korcyl, K. Kordas, A. Korn, A. A. Korol, I. Korolkov, E. V. Korolkova, O. Kortner, S. Kortner, T. Kosek, V. V. Kostyukhin, A. Kotwal, A. Kourkoumeli-Charalampidi, C. Kourkoumelis, V. Kouskoura, A. B. Kowalewska, R. Kowalewski, T. Z. Kowalski, C. Kozakai, W. Kozanecki, A. S. Kozhin, V. A. Kramarenko, G. Kramberger, D. Krasnopevtsev, M. W. Krasny, A. Krasznahorkay, A. Kravchenko, M. Kretz, J. Kretzschmar, K. Kreutzfeldt, P. Krieger, K. Krizka, K. Kroeninger, H. Kroha, J. Kroll, J. Kroseberg, J. Krstic, U. Kruchonak, H. Krüger, N. Krumnack, A. Kruse, M. C. Kruse, M. Kruskal, T. Kubota, H. Kucuk, S. Kuday, J. T. Kuechler, S. Kuehn, A. Kugel, F. Kuger, A. Kuhl, T. Kuhl, V. Kukhtin, R. Kukla, Y. Kulchitsky, S. Kuleshov, M. Kuna, T. Kunigo, A. Kupco, H. Kurashige, Y. A. Kurochkin, V. Kus, E. S. Kuwertz, M. Kuze, J. Kvita, T. Kwan, D. Kyriazopoulos, A. La Rosa, J. L. La Rosa Navarro, L. La Rotonda, C. Lacasta, F. Lacava, J. Lacey, H. Lacker, D. Lacour, V. R. Lacuesta, E. Ladygin, R. Lafaye, B. Laforge, T. Lagouri, S. Lai, S. Lammers, W. Lampl, E. Lançon, U. Landgraf, M. P. J. Landon, M. C. Lanfermann, V. S. Lang, J. C. Lange, A. J. Lankford, F. Lanni, K. Lantzsch, A. Lanza, S. Laplace, C. Lapoire, J. F. Laporte, T. Lari, F. Lasagni Manghi, M. Lassnig, P. Laurelli, W. Lavrijsen, A. T. Law, P. Laycock, T. Lazovich, M. Lazzaroni, B. Le, O. Le Dortz, E. Le Guirriec, E. P. Le Quilleuc, M. LeBlanc, T. LeCompte, F. Ledroit-Guillon, C. A. Lee, S. C. Lee, L. Lee, B. Lefebvre, G. Lefebvre, M. Lefebvre, F. Legger, C. Leggett, A. Lehan, G. Lehmann Miotto, X. Lei, W. A. Leight, A. Leisos, A. G. Leister, M. A. L. Leite, R. Leitner, D. Lellouch, B. Lemmer, K. J. C. Leney, T. Lenz, B. Lenzi, R. Leone, S. Leone, C. Leonidopoulos, S. Leontsinis, G. Lerner, C. Leroy, A. A. J. Lesage, C. G. Lester, M. Levchenko, J. Levêque, D. Levin, L. J. Levinson, M. Levy, D. Lewis, A. M. Leyko, M. Leyton, B. Li, C. Li, H. Li, H. L. Li, L. Li, L. Li, Q. Li, S. Li, X. Li, Y. Li, Z. Liang, B. Liberti, A. Liblong, P. Lichard, K. Lie, J. Liebal, W. Liebig, A. Limosani, S. C. Lin, T. H. Lin, B. E. Lindquist, A. E. Lionti, E. Lipeles, A. Lipniacka, M. Lisovyi, T. M. Liss, A. Lister, A. M. Litke, B. Liu, D. Liu, H. Liu, H. Liu, J. Liu, J. B. Liu, K. Liu, L. Liu, M. Liu, M. Liu, Y. L. Liu, Y. Liu, M. Livan, A. Lleres, J. Llorente Merino, S. L. Lloyd, F. Lo Sterzo, E. Lobodzinska, P. Loch, W. S. Lockman, F. K. Loebinger, A. E. Loevschall-Jensen, K. M. Loew, A. Loginov, T. Lohse, K. Lohwasser, M. Lokajicek, B. A. Long, J. D. Long, R. E. Long, L. Longo, K. A. Looper, L. Lopes, D. Lopez Mateos, B. Lopez Paredes, I. Lopez Paz, A. Lopez Solis, J. Lorenz, N. Lorenzo Martinez, M. Losada, P. J. Lösel, X. Lou, A. Lounis, J. Love, P. A. Love, H. Lu, N. Lu, H. J. Lubatti, C. Luci, A. Lucotte, C. Luedtke, F. Luehring, W. Lukas, L. Luminari, O. Lundberg, B. Lund-Jensen, P. M. Luzi, D. Lynn, R. Lysak, E. Lytken, V. Lyubushkin, H. Ma, L. L. Ma, Y. Ma, G. Maccarrone, A. Macchiolo, C. M. Macdonald, B. Maček, J. Machado Miguens, D. Madaffari, R. Madar, H. J. Maddocks, W. F. Mader, A. Madsen, J. Maeda, S. Maeland, T. Maeno, A. Maevskiy, E. Magradze, J. Mahlstedt, C. Maiani, C. Maidantchik, A. A. Maier, T. Maier, A. Maio, S. Majewski, Y. Makida, N. Makovec, B. Malaescu, Pa. Malecki, V. P. Maleev, F. Malek, U. Mallik, D. Malon, C. Malone, S. Maltezos, S. Malyukov, J. Mamuzic, G. Mancini, B. Mandelli, L. Mandelli, I. Mandić, J. Maneira, L. Manhaes de Andrade Filho, J. Manjarres Ramos, A. Mann, A. Manousos, B. Mansoulie, J. D. Mansour, R. Mantifel, M. Mantoani, S. Manzoni, L. Mapelli, G. Marceca, L. March, G. Marchiori, M. Marcisovsky, M. Marjanovic, D. E. Marley, F. Marroquim, S. P. Marsden, Z. Marshall, S. Marti-Garcia, B. Martin, T. A. Martin, V. J. Martin, B. Martin dit Latour, M. Martinez, V. I. Martinez Outschoorn, S. Martin-Haugh, V. S. Martoiu, A. C. Martyniuk, M. Marx, A. Marzin, L. Masetti, T. Mashimo, R. Mashinistov, J. Masik, A. L. Maslennikov, I. Massa, L. Massa, P. Mastrandrea, A. Mastroberardino, T. Masubuchi, P. Mättig, J. Mattmann, J. Maurer, S. J. Maxfield, D. A. Maximov, R. Mazini, S. M. Mazza, N. C. Mc Fadden, G. Mc Goldrick, S. P. Mc Kee, A. McCarn, R. L. McCarthy, T. G. McCarthy, L. I. McClymont, E. F. McDonald, J. A. Mcfayden, G. Mchedlidze, S. J. McMahon, R. A. McPherson, M. Medinnis, S. Meehan, S. Mehlhase, A. Mehta, K. Meier, C. Meineck, B. Meirose, D. Melini, B. R. Mellado Garcia, M. Melo, F. Meloni, A. Mengarelli, S. Menke, E. Meoni, S. Mergelmeyer, P. Mermod, L. Merola, C. Meroni, F. S. Merritt, A. Messina, J. Metcalfe, A. S. Mete, C. Meyer, C. Meyer, J.-P. Meyer, J. Meyer, H. Meyer Zu Theenhausen, F. Miano, R. P. Middleton, S. Miglioranzi, L. Mijović, G. Mikenberg, M. Mikestikova, M. Mikuž, M. Milesi, A. Milic, D. W. Miller, C. Mills, A. Milov, D. A. Milstead, A. A. Minaenko, Y. Minami, I. A. Minashvili, A. I. Mincer, B. Mindur, M. Mineev, Y. Ming, L. M. Mir, K. P. Mistry, T. Mitani, J. Mitrevski, V. A. Mitsou, A. Miucci, P. S. Miyagawa, J. U. Mjörnmark, T. Moa, K. Mochizuki, S. Mohapatra, S. Molander, R. Moles-Valls, R. Monden, M. C. Mondragon, K. Mönig, J. Monk, E. Monnier, A. Montalbano, J. Montejo Berlingen, F. Monticelli, S. Monzani, R. W. Moore, N. Morange, D. Moreno, M. Moreno Llácer, P. Morettini, D. Mori, T. Mori, M. Morii, M. Morinaga, V. Morisbak, S. Moritz, A. K. Morley, G. Mornacchi, J. D. Morris, S. S. Mortensen, L. Morvaj, M. Mosidze, J. Moss, K. Motohashi, R. Mount, E. Mountricha, S. V. Mouraviev, E. J. W. Moyse, S. Muanza, R. D. Mudd, F. Mueller, J. Mueller, R. S. P. Mueller, T. Mueller, D. Muenstermann, P. Mullen, G. A. Mullier, F. J. Munoz Sanchez, J. A. Murillo Quijada, W. J. Murray, H. Musheghyan, M. Muškinja, A. G. Myagkov, M. Myska, B. P. Nachman, O. Nackenhorst, K. Nagai, R. Nagai, K. Nagano, Y. Nagasaka, K. Nagata, M. Nagel, E. Nagy, A. M. Nairz, Y. Nakahama, K. Nakamura, T. Nakamura, I. Nakano, H. Namasivayam, R. F. Naranjo Garcia, R. Narayan, D. I. Narrias Villar, I. Naryshkin, T. Naumann, G. Navarro, R. Nayyar, H. A. Neal, P. Yu. Nechaeva, T. J. Neep, A. Negri, M. Negrini, S. Nektarijevic, C. Nellist, A. Nelson, S. Nemecek, P. Nemethy, A. A. Nepomuceno, M. Nessi, M. S. Neubauer, M. Neumann, R. M. Neves, P. Nevski, P. R. Newman, D. H. Nguyen, T. Nguyen Manh, R. B. Nickerson, R. Nicolaidou, J. Nielsen, A. Nikiforov, V. Nikolaenko, I. Nikolic-Audit, K. Nikolopoulos, J. K. Nilsen, P. Nilsson, Y. Ninomiya, A. Nisati, R. Nisius, T. Nobe, M. Nomachi, I. Nomidis, T. Nooney, S. Norberg, M. Nordberg, N. Norjoharuddeen, O. Novgorodova, S. Nowak, M. Nozaki, L. Nozka, K. Ntekas, E. Nurse, F. Nuti, F. O’grady, D. C. O’Neil, A. A. O’Rourke, V. O’Shea, F. G. Oakham, H. Oberlack, T. Obermann, J. Ocariz, A. Ochi, I. Ochoa, J. P. Ochoa-Ricoux, S. Oda, S. Odaka, H. Ogren, A. Oh, S. H. Oh, C. C. Ohm, H. Ohman, H. Oide, H. Okawa, Y. Okumura, T. Okuyama, A. Olariu, L. F. Oleiro Seabra, S. A. Olivares Pino, D. Oliveira Damazio, A. Olszewski, J. Olszowska, A. Onofre, K. Onogi, P. U. E. Onyisi, M. J. Oreglia, Y. Oren, D. Orestano, N. Orlando, R. S. Orr, B. Osculati, R. Ospanov, G. Otero y Garzon, H. Otono, M. Ouchrif, F. Ould-Saada, A. Ouraou, K. P. Oussoren, Q. Ouyang, M. Owen, R. E. Owen, V. E. Ozcan, N. Ozturk, K. Pachal, A. Pacheco Pages, L. Pacheco Rodriguez, C. Padilla Aranda, M. Pagáčová, S. Pagan Griso, F. Paige, P. Pais, K. Pajchel, G. Palacino, S. Palazzo, S. Palestini, M. Palka, D. Pallin, E. St. Panagiotopoulou, C. E. Pandini, J. G. Panduro Vazquez, P. Pani, S. Panitkin, D. Pantea, L. Paolozzi, Th. D. Papadopoulou, K. Papageorgiou, A. Paramonov, D. Paredes Hernandez, A. J. Parker, M. A. Parker, K. A. Parker, F. Parodi, J. A. Parsons, U. Parzefall, V. R. Pascuzzi, E. Pasqualucci, S. Passaggio, Fr. Pastore, G. Pásztor, S. Pataraia, J. R. Pater, T. Pauly, J. Pearce, B. Pearson, L. E. Pedersen, M. Pedersen, S. Pedraza Lopez, R. Pedro, S. V. Peleganchuk, O. Penc, C. Peng, H. Peng, J. Penwell, B. S. Peralva, M. M. Perego, D. V. Perepelitsa, E. Perez Codina, L. Perini, H. Pernegger, S. Perrella, R. Peschke, V. D. Peshekhonov, K. Peters, R. F. Y. Peters, B. A. Petersen, T. C. Petersen, E. Petit, A. Petridis, C. Petridou, P. Petroff, E. Petrolo, M. Petrov, F. Petrucci, N. E. Pettersson, A. Peyaud, R. Pezoa, P. W. Phillips, G. Piacquadio, E. Pianori, A. Picazio, E. Piccaro, M. Piccinini, M. A. Pickering, R. Piegaia, J. E. Pilcher, A. D. Pilkington, A. W. J. Pin, M. Pinamonti, J. L. Pinfold, A. Pingel, S. Pires, H. Pirumov, M. Pitt, L. Plazak, M.-A. Pleier, V. Pleskot, E. Plotnikova, P. Plucinski, D. Pluth, R. Poettgen, L. Poggioli, D. Pohl, G. Polesello, A. Poley, A. Policicchio, R. Polifka, A. Polini, C. S. Pollard, V. Polychronakos, K. Pommès, L. Pontecorvo, B. G. Pope, G. A. Popeneciu, D. S. Popovic, A. Poppleton, S. Pospisil, K. Potamianos, I. N. Potrap, C. J. Potter, C. T. Potter, G. Poulard, J. Poveda, V. Pozdnyakov, M. E. Pozo Astigarraga, P. Pralavorio, A. Pranko, S. Prell, D. Price, L. E. Price, M. Primavera, S. Prince, K. Prokofiev, F. Prokoshin, S. Protopopescu, J. Proudfoot, M. Przybycien, D. Puddu, M. Purohit, P. Puzo, J. Qian, G. Qin, Y. Qin, A. Quadt, W. B. Quayle, M. Queitsch-Maitland, D. Quilty, S. Raddum, V. Radeka, V. Radescu, S. K. Radhakrishnan, P. Radloff, P. Rados, F. Ragusa, G. Rahal, J. A. Raine, S. Rajagopalan, M. Rammensee, C. Rangel-Smith, M. G. Ratti, F. Rauscher, S. Rave, T. Ravenscroft, I. Ravinovich, M. Raymond, A. L. Read, N. P. Readioff, M. Reale, D. M. Rebuzzi, A. Redelbach, G. Redlinger, R. Reece, K. Reeves, L. Rehnisch, J. Reichert, H. Reisin, C. Rembser, H. Ren, M. Rescigno, S. Resconi, O. L. Rezanova, P. Reznicek, R. Rezvani, R. Richter, S. Richter, E. Richter-Was, O. Ricken, M. Ridel, P. Rieck, C. J. Riegel, J. Rieger, O. Rifki, M. Rijssenbeek, A. Rimoldi, M. Rimoldi, L. Rinaldi, B. Ristić, E. Ritsch, I. Riu, F. Rizatdinova, E. Rizvi, C. Rizzi, S. H. Robertson, A. Robichaud-Veronneau, D. Robinson, J. E. M. Robinson, A. Robson, C. Roda, Y. Rodina, A. Rodriguez Perez, D. Rodriguez Rodriguez, S. Roe, C. S. Rogan, O. Røhne, A. Romaniouk, M. Romano, S. M. Romano Saez, E. Romero Adam, N. Rompotis, M. Ronzani, L. Roos, E. Ros, S. Rosati, K. Rosbach, P. Rose, O. Rosenthal, N.-A. Rosien, V. Rossetti, E. Rossi, L. P. Rossi, J. H. N. Rosten, R. Rosten, M. Rotaru, I. Roth, J. Rothberg, D. Rousseau, C. R. Royon, A. Rozanov, Y. Rozen, X. Ruan, F. Rubbo, M. S. Rudolph, F. Rühr, A. Ruiz-Martinez, Z. Rurikova, N. A. Rusakovich, A. Ruschke, H. L. Russell, J. P. Rutherfoord, N. Ruthmann, Y. F. Ryabov, M. Rybar, G. Rybkin, S. Ryu, A. Ryzhov, G. F. Rzehorz, A. F. Saavedra, G. Sabato, S. Sacerdoti, H. F.-W. Sadrozinski, R. Sadykov, F. Safai Tehrani, P. Saha, M. Sahinsoy, M. Saimpert, T. Saito, H. Sakamoto, Y. Sakurai, G. Salamanna, A. Salamon, J. E. Salazar Loyola, D. Salek, P. H. Sales De Bruin, D. Salihagic, A. Salnikov, J. Salt, D. Salvatore, F. Salvatore, A. Salvucci, A. Salzburger, D. Sammel, D. Sampsonidis, A. Sanchez, J. Sánchez, V. Sanchez Martinez, H. Sandaker, R. L. Sandbach, H. G. Sander, M. Sandhoff, C. Sandoval, R. Sandstroem, D. P. C. Sankey, M. Sannino, A. Sansoni, C. Santoni, R. Santonico, H. Santos, I. Santoyo Castillo, K. Sapp, A. Sapronov, J. G. Saraiva, B. Sarrazin, O. Sasaki, Y. Sasaki, K. Sato, G. Sauvage, E. Sauvan, G. Savage, P. Savard, N. Savic, C. Sawyer, L. Sawyer, J. Saxon, C. Sbarra, A. Sbrizzi, T. Scanlon, D. A. Scannicchio, M. Scarcella, V. Scarfone, J. Schaarschmidt, P. Schacht, B. M. Schachtner, D. Schaefer, L. Schaefer, R. Schaefer, J. Schaeffer, S. Schaepe, S. Schaetzel, U. Schäfer, A. C. Schaffer, D. Schaile, R. D. Schamberger, V. Scharf, V. A. Schegelsky, D. Scheirich, M. Schernau, C. Schiavi, S. Schier, C. Schillo, M. Schioppa, S. Schlenker, K. R. Schmidt-Sommerfeld, K. Schmieden, C. Schmitt, S. Schmitt, S. Schmitz, B. Schneider, U. Schnoor, L. Schoeffel, A. Schoening, B. D. Schoenrock, E. Schopf, M. Schott, J. Schovancova, S. Schramm, M. Schreyer, N. Schuh, A. Schulte, M. J. Schultens, H.-C. Schultz-Coulon, H. Schulz, M. Schumacher, B. A. Schumm, Ph. Schune, A. Schwartzman, T. A. Schwarz, H. Schweiger, Ph. Schwemling, R. Schwienhorst, J. Schwindling, T. Schwindt, G. Sciolla, F. Scuri, F. Scutti, J. Searcy, P. Seema, S. C. Seidel, A. Seiden, F. Seifert, J. M. Seixas, G. Sekhniaidze, K. Sekhon, S. J. Sekula, D. M. Seliverstov, N. Semprini-Cesari, C. Serfon, L. Serin, L. Serkin, M. Sessa, R. Seuster, H. Severini, T. Sfiligoj, F. Sforza, A. Sfyrla, E. Shabalina, N. W. Shaikh, L. Y. Shan, R. Shang, J. T. Shank, M. Shapiro, P. B. Shatalov, K. Shaw, S. M. Shaw, A. Shcherbakova, C. Y. Shehu, P. Sherwood, L. Shi, S. Shimizu, C. O. Shimmin, M. Shimojima, M. Shiyakova, A. Shmeleva, D. Shoaleh Saadi, M. J. Shochet, S. Shojaii, S. Shrestha, E. Shulga, M. A. Shupe, P. Sicho, A. M. Sickles, P. E. Sidebo, O. Sidiropoulou, D. Sidorov, A. Sidoti, F. Siegert, Dj. Sijacki, J. Silva, S. B. Silverstein, V. Simak, Lj. Simic, S. Simion, E. Simioni, B. Simmons, D. Simon, M. Simon, P. Sinervo, N. B. Sinev, M. Sioli, G. Siragusa, S. Yu. Sivoklokov, J. Sjölin, M. B. Skinner, H. P. Skottowe, P. Skubic, M. Slater, T. Slavicek, M. Slawinska, K. Sliwa, R. Slovak, V. Smakhtin, B. H. Smart, L. Smestad, J. Smiesko, S. Yu. Smirnov, Y. Smirnov, L. N. Smirnova, O. Smirnova, M. N. K. Smith, R. W. Smith, M. Smizanska, K. Smolek, A. A. Snesarev, S. Snyder, R. Sobie, F. Socher, A. Soffer, D. A. Soh, G. Sokhrannyi, C. A. Solans Sanchez, M. Solar, E. Yu. Soldatov, U. Soldevila, A. A. Solodkov, A. Soloshenko, O. V. Solovyanov, V. Solovyev, P. Sommer, H. Son, H. Y. Song, A. Sood, A. Sopczak, V. Sopko, V. Sorin, D. Sosa, C. L. Sotiropoulou, R. Soualah, A. M. Soukharev, D. South, B. C. Sowden, S. Spagnolo, M. Spalla, M. Spangenberg, F. Spanò, D. Sperlich, F. Spettel, R. Spighi, G. Spigo, L. A. Spiller, M. Spousta, R. D. St. Denis, A. Stabile, R. Stamen, S. Stamm, E. Stanecka, R. W. Stanek, C. Stanescu, M. Stanescu-Bellu, M. M. Stanitzki, S. Stapnes, E. A. Starchenko, G. H. Stark, J. Stark, P. Staroba, P. Starovoitov, S. Stärz, R. Staszewski, P. Steinberg, B. Stelzer, H. J. Stelzer, O. Stelzer-Chilton, H. Stenzel, G. A. Stewart, J. A. Stillings, M. C. Stockton, M. Stoebe, G. Stoicea, P. Stolte, S. Stonjek, A. R. Stradling, A. Straessner, M. E. Stramaglia, J. Strandberg, S. Strandberg, A. Strandlie, M. Strauss, P. Strizenec, R. Ströhmer, D. M. Strom, R. Stroynowski, A. Strubig, S. A. Stucci, B. Stugu, N. A. Styles, D. Su, J. Su, S. Suchek, Y. Sugaya, M. Suk, V. V. Sulin, S. Sultansoy, T. Sumida, S. Sun, X. Sun, J. E. Sundermann, K. Suruliz, G. Susinno, M. R. Sutton, S. Suzuki, M. Svatos, M. Swiatlowski, I. Sykora, T. Sykora, D. Ta, C. Taccini, K. Tackmann, J. Taenzer, A. Taffard, R. Tafirout, N. Taiblum, H. Takai, R. Takashima, T. Takeshita, Y. Takubo, M. Talby, A. A. Talyshev, K. G. Tan, J. Tanaka, M. Tanaka, R. Tanaka, S. Tanaka, B. B. Tannenwald, S. Tapia Araya, S. Tapprogge, S. Tarem, G. F. Tartarelli, P. Tas, M. Tasevsky, T. Tashiro, E. Tassi, A. Tavares Delgado, Y. Tayalati, A. C. Taylor, G. N. Taylor, P. T. E. Taylor, W. Taylor, F. A. Teischinger, P. Teixeira-Dias, K. K. Temming, D. Temple, H. Ten Kate, P. K. Teng, J. J. Teoh, F. Tepel, S. Terada, K. Terashi, J. Terron, S. Terzo, M. Testa, R. J. Teuscher, T. Theveneaux-Pelzer, J. P. Thomas, J. Thomas-Wilsker, E. N. Thompson, P. D. Thompson, A. S. Thompson, L. A. Thomsen, E. Thomson, M. Thomson, M. J. Tibbetts, R. E. Ticse Torres, V. O. Tikhomirov, Yu. A. Tikhonov, S. Timoshenko, P. Tipton, S. Tisserant, K. Todome, T. Todorov, S. Todorova-Nova, J. Tojo, S. Tokár, K. Tokushuku, E. Tolley, L. Tomlinson, M. Tomoto, L. Tompkins, K. Toms, B. Tong, E. Torrence, H. Torres, E. Torró Pastor, J. Toth, F. Touchard, D. R. Tovey, T. Trefzger, A. Tricoli, I. M. Trigger, S. Trincaz-Duvoid, M. F. Tripiana, W. Trischuk, B. Trocmé, A. Trofymov, C. Troncon, M. Trottier-McDonald, M. Trovatelli, L. Truong, M. Trzebinski, A. Trzupek, J. C.-L. Tseng, P. V. Tsiareshka, G. Tsipolitis, N. Tsirintanis, S. Tsiskaridze, V. Tsiskaridze, E. G. Tskhadadze, K. M. Tsui, I. I. Tsukerman, V. Tsulaia, S. Tsuno, D. Tsybychev, Y. Tu, A. Tudorache, V. Tudorache, A. N. Tuna, S. A. Tupputi, S. Turchikhin, D. Turecek, D. Turgeman, R. Turra, A. J. Turvey, P. M. Tuts, M. Tyndel, G. Ucchielli, I. Ueda, M. Ughetto, F. Ukegawa, G. Unal, A. Undrus, G. Unel, F. C. Ungaro, Y. Unno, C. Unverdorben, J. Urban, P. Urquijo, P. Urrejola, G. Usai, A. Usanova, L. Vacavant, V. Vacek, B. Vachon, C. Valderanis, E. Valdes Santurio, N. Valencic, S. Valentinetti, A. Valero, L. Valery, S. Valkar, J. A. Valls Ferrer, W. Van Den Wollenberg, P. C. Van Der Deijl, H. van der Graaf, N. van Eldik, P. van Gemmeren, J. Van Nieuwkoop, I. van Vulpen, M. C. van Woerden, M. Vanadia, W. Vandelli, R. Vanguri, A. Vaniachine, P. Vankov, G. Vardanyan, R. Vari, E. W. Varnes, T. Varol, D. Varouchas, A. Vartapetian, K. E. Varvell, J. G. Vasquez, F. Vazeille, T. Vazquez Schroeder, J. Veatch, V. Veeraraghavan, L. M. Veloce, F. Veloso, S. Veneziano, A. Ventura, M. Venturi, N. Venturi, A. Venturini, V. Vercesi, M. Verducci, W. Verkerke, J. C. Vermeulen, A. Vest, M. C. Vetterli, O. Viazlo, I. Vichou, T. Vickey, O. E. Vickey Boeriu, G. H. A. Viehhauser, S. Viel, L. Vigani, M. Villa, M. Villaplana Perez, E. Vilucchi, M. G. Vincter, V. B. Vinogradov, C. Vittori, I. Vivarelli, S. Vlachos, M. Vlasak, M. Vogel, P. Vokac, G. Volpi, M. Volpi, H. von der Schmitt, E. von Toerne, V. Vorobel, K. Vorobev, M. Vos, R. Voss, J. H. Vossebeld, N. Vranjes, M. Vranjes Milosavljevic, V. Vrba, M. Vreeswijk, R. Vuillermet, I. Vukotic, Z. Vykydal, P. Wagner, W. Wagner, H. Wahlberg, S. Wahrmund, J. Wakabayashi, J. Walder, R. Walker, W. Walkowiak, V. Wallangen, C. Wang, C. Wang, F. Wang, H. Wang, H. Wang, J. Wang, J. Wang, K. Wang, R. Wang, S. M. Wang, T. Wang, T. Wang, W. Wang, X. Wang, C. Wanotayaroj, A. Warburton, C. P. Ward, D. R. Wardrope, A. Washbrook, P. M. Watkins, A. T. Watson, M. F. Watson, G. Watts, S. Watts, B. M. Waugh, S. Webb, M. S. Weber, S. W. Weber, J. S. Webster, A. R. Weidberg, B. Weinert, J. Weingarten, C. Weiser, H. Weits, P. S. Wells, T. Wenaus, T. Wengler, S. Wenig, N. Wermes, M. Werner, M. D. Werner, P. Werner, M. Wessels, J. Wetter, K. Whalen, N. L. Whallon, A. M. Wharton, A. White, M. J. White, R. White, D. Whiteson, F. J. Wickens, W. Wiedenmann, M. Wielers, P. Wienemann, C. Wiglesworth, L. A. M. Wiik-Fuchs, A. Wildauer, F. Wilk, H. G. Wilkens, H. H. Williams, S. Williams, C. Willis, S. Willocq, J. A. Wilson, I. Wingerter-Seez, F. Winklmeier, O. J. Winston, B. T. Winter, M. Wittgen, J. Wittkowski, T. M. H. Wolf, M. W. Wolter, H. Wolters, S. D. Worm, B. K. Wosiek, J. Wotschack, M. J. Woudstra, K. W. Wozniak, M. Wu, M. Wu, S. L. Wu, X. Wu, Y. Wu, T. R. Wyatt, B. M. Wynne, S. Xella, D. Xu, L. Xu, B. Yabsley, S. Yacoob, D. Yamaguchi, Y. Yamaguchi, A. Yamamoto, S. Yamamoto, T. Yamanaka, K. Yamauchi, Y. Yamazaki, Z. Yan, H. Yang, H. Yang, Y. Yang, Z. Yang, W.-M. Yao, Y. C. Yap, Y. Yasu, E. Yatsenko, K. H. Yau Wong, J. Ye, S. Ye, I. Yeletskikh, A. L. Yen, E. Yildirim, K. Yorita, R. Yoshida, K. Yoshihara, C. Young, C. J. S. Young, S. Youssef, D. R. Yu, J. Yu, J. M. Yu, J. Yu, L. Yuan, S. P. Y. Yuen, I. Yusuff, B. Zabinski, R. Zaidan, A. M. Zaitsev, N. Zakharchuk, J. Zalieckas, A. Zaman, S. Zambito, L. Zanello, D. Zanzi, C. Zeitnitz, M. Zeman, A. Zemla, J. C. Zeng, Q. Zeng, K. Zengel, O. Zenin, T. Ženiš, D. Zerwas, D. Zhang, F. Zhang, G. Zhang, H. Zhang, J. Zhang, L. Zhang, R. Zhang, R. Zhang, X. Zhang, Z. Zhang, X. Zhao, Y. Zhao, Z. Zhao, A. Zhemchugov, J. Zhong, B. Zhou, C. Zhou, L. Zhou, L. Zhou, M. Zhou, N. Zhou, C. G. Zhu, H. Zhu, J. Zhu, Y. Zhu, X. Zhuang, K. Zhukov, A. Zibell, D. Zieminska, N. I. Zimine, C. Zimmermann, S. Zimmermann, Z. Zinonos, M. Zinser, M. Ziolkowski, L. Živković, G. Zobernig, A. Zoccoli, M. zur Nedden, L. Zwalinski, ATLAS Collaboration

**Affiliations:** 1 Department of Physics, University of Adelaide, Adelaide, Australia; 2 Physics Department, SUNY Albany, Albany, NY USA; 3 Department of Physics, University of Alberta, Edmonton, AB Canada; 4 Department of Physics, Ankara University, Ankara, Turkey; 5 Istanbul Aydin University, Istanbul, Turkey; 6 Division of Physics, TOBB University of Economics and Technology, Ankara, Turkey; 7 LAPP, CNRS/IN2P3 and Université Savoie Mont Blanc, Annecy-le-Vieux, France; 8 High Energy Physics Division, Argonne National Laboratory, Argonne, IL USA; 9 Department of Physics, University of Arizona, Tucson, AZ USA; 10 Department of Physics, The University of Texas at Arlington, Arlington, TX USA; 11 Physics Department, University of Athens, Athens, Greece; 12 Budker Institute of Nuclear Physics, SB RAS, Novosibirsk, Russia; 13 Department of Physics, The University of Texas at Austin, Austin, TX USA; 14 Institute of Physics, Azerbaijan Academy of Sciences, Baku, Azerbaijan; 15 Institut de Física d’Altes Energies (IFAE), The Barcelona Institute of Science and Technology, Barcelona, Spain; 16 Institute of Physics, University of Belgrade, Belgrade, Serbia; 17 Department for Physics and Technology, University of Bergen, Bergen, Norway; 18 Physics Division, Lawrence Berkeley National Laboratory and University of California, Berkeley, CA USA; 19 Department of Physics, Humboldt University, Berlin, Germany; 20 Albert Einstein Center for Fundamental Physics and Laboratory for High Energy Physics, University of Bern, Bern, Switzerland; 21 School of Physics and Astronomy, University of Birmingham, Birmingham, UK; 22 Department of Physics, Bogazici University, Istanbul, Turkey; 23 Department of Physics Engineering, Gaziantep University, Gaziantep, Turkey; 24 Faculty of Engineering and Natural Sciences, Istanbul Bilgi University, Istanbul, Turkey; 25 Faculty of Engineering and Natural Sciences, Bahcesehir University, Istanbul, Turkey; 26 Centro de Investigaciones, Universidad Antonio Narino, Bogotá, Colombia; 27 INFN Sezione di Bologna, Bologna, Italy; 28 Dipartimento di Fisica e Astronomia, Università di Bologna, Bologna, Italy; 29 Physikalisches Institut, University of Bonn, Bonn, Germany; 30 Department of Physics, Boston University, Boston, MA USA; 31 Department of Physics, Brandeis University, Waltham, MA USA; 32 Universidade Federal do Rio De Janeiro COPPE/EE/IF, Rio de Janeiro, Brazil; 33 Electrical Circuits Department, Federal University of Juiz de Fora (UFJF), Juiz de Fora, Brazil; 34 Federal University of Sao Joao del Rei (UFSJ), Sao Joao del Rei, Brazil; 35 Instituto de Fisica, Universidade de Sao Paulo, São Paulo, Brazil; 36 Physics Department, Brookhaven National Laboratory, Upton, NY USA; 37 Transilvania University of Brasov, Brasov, Romania; 38 National Institute of Physics and Nuclear Engineering, Bucharest, Romania; 39 Physics Department, National Institute for Research and Development of Isotopic and Molecular Technologies, Cluj Napoca, Romania; 40 University Politehnica Bucharest, Bucharest, Romania; 41 West University in Timisoara, Timisoara, Romania; 42 Departamento de Física, Universidad de Buenos Aires, Buenos Aires, Argentina; 43 Cavendish Laboratory, University of Cambridge, Cambridge, UK; 44 Department of Physics, Carleton University, Ottawa, ON Canada; 45 CERN, Geneva, Switzerland; 46 Enrico Fermi Institute, University of Chicago, Chicago, IL USA; 47 Departamento de Física, Pontificia Universidad Católica de Chile, Santiago, Chile; 48 Departamento de Física, Universidad Técnica Federico Santa María, Valparaiso, Chile; 49 Institute of High Energy Physics, Chinese Academy of Sciences, Beijing, China; 50 Department of Modern Physics, University of Science and Technology of China, Hefei, Anhui China; 51 Department of Physics, Nanjing University, Nanjing, Jiangsu China; 52 School of Physics, Shandong University, Jinan, Shandong China; 53 Shanghai Key Laboratory for Particle Physics and Cosmology, Department of Physics and Astronomy, Shanghai Jiao Tong University (also affiliated with PKU-CHEP), Shanghai, China; 54 Physics Department, Tsinghua University, Beijing, 100084 China; 55 Laboratoire de Physique Corpusculaire, Clermont Université and Université Blaise Pascal and CNRS/IN2P3, Clermont-Ferrand, France; 56 Nevis Laboratory, Columbia University, Irvington, NY USA; 57 Niels Bohr Institute, University of Copenhagen, Kobenhavn, Denmark; 58 INFN Gruppo Collegato di Cosenza, Laboratori Nazionali di Frascati, Frascati, Italy; 59 Dipartimento di Fisica, Università della Calabria, Rende, Italy; 60 Faculty of Physics and Applied Computer Science, AGH University of Science and Technology, Kraków, Poland; 61 Marian Smoluchowski Institute of Physics, Jagiellonian University, Kraków, Poland; 62 Institute of Nuclear Physics, Polish Academy of Sciences, Kraków, Poland; 63 Physics Department, Southern Methodist University, Dallas, TX USA; 64 Physics Department, University of Texas at Dallas, Richardson, TX USA; 65 DESY, Hamburg, Zeuthen, Germany; 66 Lehrstuhl für Experimentelle Physik IV, Technische Universität Dortmund, Dortmund, Germany; 67 Institut für Kern- und Teilchenphysik, Technische Universität Dresden, Dresden, Germany; 68 Department of Physics, Duke University, Durham, NC USA; 69 SUPA-School of Physics and Astronomy, University of Edinburgh, Edinburgh, UK; 70 INFN Laboratori Nazionali di Frascati, Frascati, Italy; 71 Fakultät für Mathematik und Physik, Albert-Ludwigs-Universität, Freiburg, Germany; 72 Section de Physique, Université de Genève, Geneva, Switzerland; 73 INFN Sezione di Genova, Genoa, Italy; 74 Dipartimento di Fisica, Università di Genova, Genoa, Italy; 75 E. Andronikashvili Institute of Physics, Iv. Javakhishvili Tbilisi State University, Tbilisi, Georgia; 76 High Energy Physics Institute, Tbilisi State University, Tbilisi, Georgia; 77 II Physikalisches Institut, Justus-Liebig-Universität Giessen, Giessen, Germany; 78 SUPA-School of Physics and Astronomy, University of Glasgow, Glasgow, UK; 79 II Physikalisches Institut, Georg-August-Universität, Göttingen, Germany; 80 Laboratoire de Physique Subatomique et de Cosmologie, Université Grenoble-Alpes, CNRS/IN2P3, Grenoble, France; 81 Laboratory for Particle Physics and Cosmology, Harvard University, Cambridge, MA USA; 82 Kirchhoff-Institut für Physik, Ruprecht-Karls-Universität Heidelberg, Heidelberg, Germany; 83 Physikalisches Institut, Ruprecht-Karls-Universität Heidelberg, Heidelberg, Germany; 84 ZITI Institut für technische Informatik, Ruprecht-Karls-Universität Heidelberg, Mannheim, Germany; 85 Faculty of Applied Information Science, Hiroshima Institute of Technology, Hiroshima, Japan; 86 Department of Physics, The Chinese University of Hong Kong, Shatin, N.T. Hong Kong; 87 Department of Physics, The University of Hong Kong, Pok Fu Lam, Hong Kong; 88 Department of Physics, The Hong Kong University of Science and Technology, Clear Water Bay, Kowloon, Hong Kong, China; 89 Department of Physics, Indiana University, Bloomington, IN USA; 90 Institut für Astro- und Teilchenphysik, Leopold-Franzens-Universität, Innsbruck, Austria; 91 University of Iowa, Iowa City, IA USA; 92 Department of Physics and Astronomy, Iowa State University, Ames, IA USA; 93 Joint Institute for Nuclear Research, JINR Dubna, Dubna, Russia; 94 KEK, High Energy Accelerator Research Organization, Tsukuba, Japan; 95 Graduate School of Science, Kobe University, Kobe, Japan; 96 Faculty of Science, Kyoto University, Kyoto, Japan; 97 Kyoto University of Education, Kyoto, Japan; 98 Department of Physics, Kyushu University, Fukuoka, Japan; 99 Instituto de Física La Plata, Universidad Nacional de La Plata and CONICET, La Plata, Argentina; 100 Physics Department, Lancaster University, Lancaster, UK; 101 INFN Sezione di Lecce, Lecce, Italy; 102 Dipartimento di Matematica e Fisica, Università del Salento, Lecce, Italy; 103 Oliver Lodge Laboratory, University of Liverpool, Liverpool, UK; 104 Department of Physics, Jožef Stefan Institute and University of Ljubljana, Ljubljana, Slovenia; 105 School of Physics and Astronomy, Queen Mary University of London, London, UK; 106 Department of Physics, Royal Holloway University of London, Surrey, UK; 107 Department of Physics and Astronomy, University College London, London, UK; 108 Louisiana Tech University, Ruston, LA USA; 109 Laboratoire de Physique Nucléaire et de Hautes Energies, UPMC and Université Paris-Diderot and CNRS/IN2P3, Paris, France; 110 Fysiska Institutionen, Lunds Universitet, Lund, Sweden; 111 Departamento de Fisica Teorica C-15, Universidad Autonoma de Madrid, Madrid, Spain; 112 Institut für Physik, Universität Mainz, Mainz, Germany; 113 School of Physics and Astronomy, University of Manchester, Manchester, UK; 114 CPPM, Aix-Marseille Université and CNRS/IN2P3, Marseille, France; 115 Department of Physics, University of Massachusetts, Amherst, MA USA; 116 Department of Physics, McGill University, Montreal, QC Canada; 117 School of Physics, University of Melbourne, Melbourne, VIC Australia; 118 Department of Physics, The University of Michigan, Ann Arbor, MI USA; 119 Department of Physics and Astronomy, Michigan State University, East Lansing, MI USA; 120 INFN Sezione di Milano, Milan, Italy; 121 Dipartimento di Fisica, Università di Milano, Milan, Italy; 122 B.I. Stepanov Institute of Physics, National Academy of Sciences of Belarus, Minsk, Republic of Belarus; 123 National Scientific and Educational Centre for Particle and High Energy Physics, Minsk, Republic of Belarus; 124 Group of Particle Physics, University of Montreal, Montreal, QC Canada; 125 P.N. Lebedev Physical Institute of the Russian, Academy of Sciences, Moscow, Russia; 126 Institute for Theoretical and Experimental Physics (ITEP), Moscow, Russia; 127 National Research Nuclear University MEPhI, Moscow, Russia; 128 D.V. Skobeltsyn Institute of Nuclear Physics, M.V. Lomonosov Moscow State University, Moscow, Russia; 129 Fakultät für Physik, Ludwig-Maximilians-Universität München, Munich, Germany; 130 Max-Planck-Institut für Physik (Werner-Heisenberg-Institut), Munich, Germany; 131 Nagasaki Institute of Applied Science, Nagasaki, Japan; 132 Graduate School of Science and Kobayashi-Maskawa Institute, Nagoya University, Nagoya, Japan; 133 INFN Sezione di Napoli, Naples, Italy; 134 Dipartimento di Fisica, Università di Napoli, Naples, Italy; 135 Department of Physics and Astronomy, University of New Mexico, Albuquerque, NM USA; 136 Institute for Mathematics, Astrophysics and Particle Physics, Radboud University Nijmegen/Nikhef, Nijmegen, The Netherlands; 137 Nikhef National Institute for Subatomic Physics and University of Amsterdam, Amsterdam, The Netherlands; 138 Department of Physics, Northern Illinois University, DeKalb, IL USA; 139 Budker Institute of Nuclear Physics, SB RAS, Novosibirsk, Russia; 140 Department of Physics, New York University, New York, NY USA; 141 Ohio State University, Columbus, OH USA; 142 Faculty of Science, Okayama University, Okayama, Japan; 143 Homer L. Dodge Department of Physics and Astronomy, University of Oklahoma, Norman, OK USA; 144 Department of Physics, Oklahoma State University, Stillwater, OK USA; 145 Palacký University, RCPTM, Olomouc, Czech Republic; 146 Center for High Energy Physics, University of Oregon, Eugene, OR USA; 147 LAL, University of Paris-Sud, CNRS/IN2P3, Université Paris-Saclay, Orsay, France; 148 Graduate School of Science, Osaka University, Osaka, Japan; 149 Department of Physics, University of Oslo, Oslo, Norway; 150 Department of Physics, Oxford University, Oxford, UK; 151 INFN Sezione di Pavia, Pavia, Italy; 152 Dipartimento di Fisica, Università di Pavia, Pavia, Italy; 153 Department of Physics, University of Pennsylvania, Philadelphia, PA USA; 154 National Research Centre “Kurchatov Institute” B.P. Konstantinov Petersburg Nuclear Physics Institute, St. Petersburg, Russia; 155 INFN Sezione di Pisa, Pisa, Italy; 156 Dipartimento di Fisica E. Fermi, Università di Pisa, Pisa, Italy; 157 Department of Physics and Astronomy, University of Pittsburgh, Pittsburgh, PA USA; 158 Laboratório de Instrumentação e Física Experimental de Partículas-LIP, Lisbon, Portugal; 159 Faculdade de Ciências, Universidade de Lisboa, Lisbon, Portugal; 160 Department of Physics, University of Coimbra, Coimbra, Portugal; 161 Centro de Física Nuclear da Universidade de Lisboa, Lisbon, Portugal; 162 Departamento de Fisica, Universidade do Minho, Braga, Portugal; 163 Departamento de Fisica Teorica y del Cosmos and CAFPE, Universidad de Granada, Granada, Spain; 164 Dep Fisica and CEFITEC of Faculdade de Ciencias e Tecnologia, Universidade Nova de Lisboa, Caparica, Portugal; 165 Institute of Physics, Academy of Sciences of the Czech Republic, Prague, Czech Republic; 166 Czech Technical University in Prague, Prague, Czech Republic; 167 Faculty of Mathematics and Physics, Charles University in Prague, Prague, Czech Republic; 168 State Research Center Institute for High Energy Physics (Protvino), NRC KI, Protvino, Russia; 169 Particle Physics Department, Rutherford Appleton Laboratory, Didcot, UK; 170 INFN Sezione di Roma Tor Vergata, Rome, Italy; 171 Dipartimento di Fisica, Università di Roma Tor Vergata, Rome, Italy; 172 INFN Sezione di Roma Tor Vergata, Rome, Italy; 173 Dipartimento di Fisica, Università di Roma Tor Vergata, Rome, Italy; 174 INFN Sezione di Roma Tre, Rome, Italy; 175 Dipartimento di Matematica e Fisica, Università Roma Tre, Rome, Italy; 176 Faculté des Sciences Ain Chock, Réseau Universitaire de Physique des Hautes Energies-Université Hassan II, Casablanca, Morocco; 177 Centre National de l’Energie des Sciences Techniques Nucleaires, Rabat, Morocco; 178 Faculté des Sciences Semlalia, Université Cadi Ayyad, LPHEA-Marrakech, Marrakech, Morocco; 179 Faculté des Sciences, Université Mohamed Premier and LPTPM, Oujda, Morocco; 180 Faculté des Sciences, Université Mohammed V, Rabat, Morocco; 181 DSM/IRFU (Institut de Recherches sur les Lois Fondamentales de l’Univers), CEA Saclay (Commissariat à l’Energie Atomique et aux Energies Alternatives), Gif-sur-Yvette, France; 182 Santa Cruz Institute for Particle Physics, University of California Santa Cruz, Santa Cruz, CA USA; 183 Department of Physics, University of Washington, Seattle, WA USA; 184 Department of Physics and Astronomy, University of Sheffield, Sheffield, UK; 185 Department of Physics, Shinshu University, Nagano, Japan; 186 Fachbereich Physik, Universität Siegen, Siegen, Germany; 187 Department of Physics, Simon Fraser University, Burnaby, BC Canada; 188 SLAC National Accelerator Laboratory, Stanford, CA USA; 189 Faculty of Mathematics, Physics and Informatics, Comenius University, Bratislava, Slovak Republic; 190 Department of Subnuclear Physics, Institute of Experimental Physics of the Slovak Academy of Sciences, Kosice, Slovak Republic; 191 Department of Physics, University of Cape Town, Cape Town, South Africa; 192 Department of Physics, University of Johannesburg, Johannesburg, South Africa; 193 School of Physics, University of the Witwatersrand, Johannesburg, South Africa; 194 Department of Physics, Stockholm University, Stockholm, Sweden; 195 The Oskar Klein Centre, Stockholm, Sweden; 196 Physics Department, Royal Institute of Technology, Stockholm, Sweden; 197 Departments of Physics and Astronomy and Chemistry, Stony Brook University, Stony Brook, NY USA; 198 Department of Physics and Astronomy, University of Sussex, Brighton, UK; 199 School of Physics, University of Sydney, Sydney, Australia; 200 Institute of Physics, Academia Sinica, Taipei, Taiwan; 201 Department of Physics, Technion: Israel Institute of Technology, Haifa, Israel; 202 Raymond and Beverly Sackler School of Physics and Astronomy, Tel Aviv University, Tel Aviv, Israel; 203 Department of Physics, Aristotle University of Thessaloniki, Thessaloníki, Greece; 204 International Center for Elementary Particle Physics and Department of Physics, The University of Tokyo, Tokyo, Japan; 205 Graduate School of Science and Technology, Tokyo Metropolitan University, Tokyo, Japan; 206 Department of Physics, Tokyo Institute of Technology, Tokyo, Japan; 207 Department of Physics, University of Toronto, Toronto, ON Canada; 208 TRIUMF, Vancouver, BC Canada; 209 Department of Physics and Astronomy, York University, Toronto, ON Canada; 210 Faculty of Pure and Applied Sciences, and Center for Integrated Research in Fundamental Science and Engineering, University of Tsukuba, Tsukuba, Japan; 211 Department of Physics and Astronomy, Tufts University, Medford, MA USA; 212 Department of Physics and Astronomy, University of California Irvine, Irvine, CA USA; 213 INFN Gruppo Collegato di Udine, Sezione di Trieste, Udine, Italy; 214 ICTP, Trieste, Italy; 215 Dipartimento di Chimica Fisica e Ambiente, Università di Udine, Udine, Italy; 216 Department of Physics and Astronomy, University of Uppsala, Uppsala, Sweden; 217 Department of Physics, University of Illinois, Urbana, IL USA; 218 Instituto de Fisica Corpuscular (IFIC) and Departamento de Fisica Atomica, Molecular y Nuclear and Departamento de Ingeniería Electrónica and Instituto de Microelectrónica de Barcelona (IMB-CNM), University of Valencia and CSIC, Valencia, Spain; 219 Department of Physics, University of British Columbia, Vancouver, BC Canada; 220 Department of Physics and Astronomy, University of Victoria, Victoria, BC Canada; 221 Department of Physics, University of Warwick, Coventry, UK; 222 Waseda University, Tokyo, Japan; 223 Department of Particle Physics, The Weizmann Institute of Science, Rehovot, Israel; 224 Department of Physics, University of Wisconsin, Madison, WI USA; 225 Fakultät für Physik und Astronomie, Julius-Maximilians-Universität, Würzburg, Germany; 226 Fakultät für Mathematik und Naturwissenschaften, Fachgruppe Physik, Bergische Universität Wuppertal, Wuppertal, Germany; 227 Department of Physics, Yale University, New Haven, CT USA; 228 Yerevan Physics Institute, Yerevan, Armenia; 229 Centre de Calcul de l’Institut National de Physique Nucléaire et de Physique des Particules (IN2P3), Villeurbanne, France

## Abstract

The result of a search for pair production of the supersymmetric partner of the Standard Model bottom quark ($\tilde{b}^{}_{1}$) is reported. The search uses 3.2 fb$^{-1}$  of *pp* collisions at $\sqrt{s}=13$ TeV collected by the ATLAS experiment at the Large Hadron Collider in 2015. Bottom squarks are searched for in events containing large missing transverse momentum and exactly two jets identified as originating from *b*-quarks. No excess above the expected Standard Model background yield is observed. Exclusion limits at 95 % confidence level on the mass of the bottom squark are derived in phenomenological supersymmetric *R*-parity-conserving models in which the $\tilde{b}^{}_{1}$ is the lightest squark and is assumed to decay exclusively via $\tilde{b}^{}_{1} \rightarrow b \tilde{\chi }^{0}_{1}$, where $\tilde{\chi }^{0}_{1}$ is the lightest neutralino. The limits significantly extend previous results; bottom squark masses up to 800 (840) GeV are excluded for the $\tilde{\chi }^{0}_{1}$ mass below 360 (100) GeV whilst differences in mass above 100 GeV between the $\tilde{b}^{}_{1}$ and the $\tilde{\chi }^{0}_{1}$ are excluded up to a $\tilde{b}^{}_{1}$ mass of 500 GeV.

## Introduction

Supersymmetry (SUSY) [1–6] provides an extension of the Standard Model (SM) that solves the hierarchy problem [7–10] by introducing supersymmetric partners of the known bosons and fermions. In the framework of the *R*-parity-conserving minimal supersymmetric extension of the SM (MSSM) [11–13], SUSY particles are produced in pairs and the lightest supersymmetric particle (LSP) is stable, providing a possible candidate for dark matter [14, 15]. In a large variety of models the LSP is the lightest neutralino ($\tilde{\chi }^{0}_{1}$). Naturalness considerations [16, 17] suggest that the supersymmetric partners of the third-generation SM quarks are the lightest coloured supersymmetric particles. This may lead to the lightest bottom squark ($\tilde{b}^{}_{1}$) and top squark ($\tilde{t}^{}_{1}$) mass eigenstates^1^ being significantly lighter than the other squarks and the gluinos. As a consequence, $\tilde{b}^{}_{1}$ and $\tilde{t}^{}_{1}$ could be pair-produced with relatively large cross-sections at the Large Hadron Collider (LHC).

This paper presents a search for the pair production of bottom squarks decaying exclusively as $\tilde{b}^{}_{1} \rightarrow b\tilde{\chi }^{0}_{1}$ using 3.2 fb$^{-1}$  of proton–proton (*pp*) collisions at $\sqrt{s}=13$ TeV collected by the ATLAS experiment at the LHC in 2015. A SUSY particle mass hierarchy with this exclusive decay has been predicted by various phenomenological MSSM models [18]. Searches with the $\sqrt{s}=8$ TeV LHC Run-1 dataset at ATLAS and CMS have set limits on $\tilde{b}^{}_{1}$ masses in such scenarios. For $\tilde{\chi }^{0}_{1}$ masses around 100 GeV, exclusion limits at 95 % confidence level (CL) up to 620 and 680 GeV have been reported by the ATLAS [19] and CMS [20] collaborations, respectively. The searches were performed in events characterized by the presence of large missing transverse momentum and two jets identified as containing *b*-hadrons ($b$-jets). In Run 2 of the LHC the production cross-section rises due to the increase of the centre-of-mass energy of the *pp* collisions. For instance, for a $\tilde{b}^{}_{1}$ with a mass of 800 GeV, the production cross-section increases by almost a factor of ten going from $\sqrt{s}=8$ TeV to $\sqrt{s}=13$ TeV. In addition, the sensitivity of the analysis benefits from the improved algorithms adopted to identify *b*-jets and use of information from the newly installed pixel layer in the Run-2 ATLAS detector. The search strategy at 13 TeV closely follows the previous ATLAS studies, with signal regions defined to provide sensitivity to the kinematic topologies arising from different mass splittings between the bottom squark and the neutralino.

## ATLAS detector

The ATLAS detector [21] is a multi-purpose particle physics detector with a forward-backward symmetric cylindrical geometry and nearly 4$\pi$ coverage in solid angle.^2^ The inner tracking detector consists of pixel and silicon microstrip detectors covering the pseudorapidity region $|\eta |<2.5$, surrounded by a transition radiation tracker which enhances electron identification in the region $|\eta |<2.0$. Between Run 1 and Run 2, a new inner pixel layer, the Insertable B-Layer (IBL) [22], was inserted at a mean sensor radius of 3.3 cm. The inner detector is surrounded by a thin superconducting solenoid providing an axial 2 T magnetic field and by a fine-granularity lead/liquid-argon (LAr) electromagnetic calorimeter covering $|\eta |<3.2$. A steel/scintillator-tile calorimeter provides hadronic coverage in the central pseudorapidity range ($|\eta |<1.7$). The endcap and forward regions ($1.5<|\eta |<4.9$) of the hadronic calorimeter are made of LAr active layers with either copper or tungsten as the absorber material. An extensive muon spectrometer with an air-core toroid magnet system surrounds the calorimeters. Three layers of high-precision tracking chambers provide coverage in the range $|\eta |<2.7$, while dedicated fast chambers allow triggering in the region $|\eta |<2.4$. The ATLAS trigger system consists of a hardware-based level-1 trigger followed by a software-based high-level trigger [23].

## Data and simulated event samples

The data used in this analysis were collected by the ATLAS detector in *pp* collisions at the LHC with a centre-of-mass energy of 13 TeV and 25 ns proton bunch crossing interval during 2015. After applying beam-, data- and detector-quality criteria, the available dataset corresponds to an integrated luminosity of 3.2 fb$^{-1}$ with an uncertainty of ±5 %. The uncertainty is derived from a calibration of the luminosity scale using a pair of *x*–*y* beam-separation scans performed in August 2015 and following a methodology similar to that detailed in Ref. [24]. In this dataset, each event includes an average of approximately 14 additional inelastic *pp* collisions in the same bunch crossing (in-time pile-up). The events used in this search were selected using a trigger logic that accepts events with an uncorrected missing transverse momentum above 70 GeV, calculated using a sum over calorimeter cells. Additional events selected with lepton- and photon-based triggers are employed for control regions defined to estimate SM background contributions. All the selections employed in this paper use a highly efficient trigger selection. The triggers used are in a plateau region and the systematic uncertainties related to the trigger simulation are found to be negligible.

Monte Carlo (MC) simulated event samples are used to model the expected signal and to aid in the description and estimation of SM background processes. The response of the detector is simulated [25] either fully by a software program based on GEANT4  [26] or by a faster simulation based on a parameterization [27] for the calorimeter response and GEANT4 for the other detector systems. To account for additional *pp* interactions from the same and close-by bunch crossings, a set of minimum-bias interactions generated using Pythia  [28] 8.186 and the MSTW2008LO [29, 30] parton distribution function (PDF) set is superimposed onto the hard scattering events to reproduce the observed distribution of the average number of interactions per bunch crossing.

The signal samples are generated using MadGraph5_aMC@NLO  [31] v2.2.3 interfaced to Pythia  8.186 with the A14 [32] set of parameters (tune) for the modelling of the parton showering (PS), hadronization and underlying event. The matrix element (ME) calculation is performed at tree level and includes the emission of up to two additional partons. The PDF set used for the generation is NNPDF23LO [33]. The ME–PS matching is done using the CKKW-L [34] prescription, with a matching scale set to one quarter of the bottom squark mass. The cross-sections used to evaluate the signal yields are calculated to next-to-leading-order accuracy in the strong coupling constant, adding the resummation of soft gluon emission at next-to-leading-logarithmic accuracy (NLO + NLL) [35–37].

SM background samples are simulated using different MC generator programs depending on the process. Events containing *W* or *Z* bosons with associated jets, including jets from the fragmentation of *b*- and *c*-quarks (heavy-flavour jets), are simulated using the Sherpa 2.1.1 [38] generator. Matrix elements are calculated for up to two additional partons at next-to-leading order (NLO) and four partons at leading order (LO) using the Comix  [39] and OpenLoops  [40] matrix element generators and merged with the Sherpa PS [41] using the ME + PS@NLO prescription [42]. The CT10 [43] PDF set is used in conjunction with a dedicated PS tune developed by the Sherpa authors. The *W*/*Z*+jets events are normalized to their next-to-NLO (NNLO) QCD theoretical cross-sections [44]. For a cross-check of the modelling of $Z$+jets background, samples of events containing a photon produced in association with jets, including heavy-flavour jets, are simulated using the Sherpa 2.1.1 generator with matrix elements calculated at LO with up to four partons for the LO calculation.

Diboson processes are also simulated using the Sherpa 2.1.1 generator with the same settings as the single-boson samples. They are calculated for up to one (*ZZ*) or zero (*WW*, *WZ*) additional partons at NLO and up to three additional partons at LO. The NLO generator cross-sections are scaled down by 9 % to account for the usage of $\alpha ^{}_\mathrm{QED}$=1/129 rather than 1/132, the latter corresponding to the use of the parameters defined by the Particle Data Group as input to the $G^{}_{\mu }$ scheme [45].

Top-quark pair and single-top-quark production (*Wt*- and *s*-channel) events are simulated using the Powheg-Box v2 [46] generator as described in Ref. [47] with the CT10 PDF set in the matrix element calculations. Electroweak *t*-channel single-top-quark events are generated using the Powheg-Box v1 generator. The top-quark mass is set to 172.5 GeV. For the $t\bar{t}$ production, the $h^{}_\mathrm{damp}$ parameter, which controls the transverse momentum ($p_{\text {T}}$) of the first additional emission beyond the Born configuration and thus regulates the $p_{\text {T}}$  of the recoil emission against the $t\bar{t}$  system, is set to the mass of the top quark. For all processes, the parton shower, fragmentation, and the underlying event are simulated using Pythia  6.428 [48] with the CTEQ6L1 PDF set and the corresponding Perugia 2012 tune (P2012) [49]. The $t\bar{t}$ samples are normalized to their NNLO cross-section including the resummation of soft gluon emission at next-to-NLL accuracy using Top++2.0 [50]. Samples of single-top-quark production are normalized to the NLO cross-sections reported in Refs. [51–53] for the *s*-, *t*- and *Wt*-channels, respectively. The associated production of a top-quark pair with a vector boson, $t\bar{t} +W/Z$, is generated at LO with MadGraph5_aMC@NLO  v2.2.3 interfaced to Pythia  8.186, with up to two ($t\bar{t} +W$), one ($t\bar{t} +Z$) or no ($t\bar{t} +W W$) extra partons included in the matrix elements. The samples are normalized to their NLO cross-sections [31].

The EvtGen 1.2.0 program [54] is used for modelling the properties of bottom- and charm-hadron decays in all samples generated with MadGraph5_aMC@NLO and Powheg-Box.

Several samples produced without detector simulation are employed to derive systematic uncertainties associated with the specific configuration of the MC generators used for the nominal SM background samples. They include variations of the renormalization and factorization scales, the CKKW-L matching scale, as well as different PDF sets and fragmentation/hadronization models. Details of the MC modelling uncertainties are discussed in Sect. 7.

## Event reconstruction

The search for bottom squark pair production is based on a selection of events with jets and large missing transverse momentum in the final state. Events containing electrons or muons are explicitly vetoed in the signal and validation regions, and are used to define control regions. An overlap removal procedure is applied to prevent double-counting of reconstructed objects. The details of the selections and overlap removal are given below.

Interaction vertices from the *pp* collisions are reconstructed from tracks in the inner detector. Events must have at least one primary reconstructed vertex, required to be consistent with the beamspot envelope and to consist of at least two tracks with $p_{\text {T}}$ > 0.4 GeV. When more than one such vertex is found the one with the largest sum of the square of transverse momenta of associated tracks [55] is chosen.

Jet candidates are reconstructed from three-dimensional energy clusters [56] in the calorimeter using the anti-$k_t$ jet algorithm [57] with a radius parameter of 0.4. Each topological cluster’s energy is calibrated to the electromagnetic scale prior to jet reconstruction. The reconstructed jets are then calibrated to the particle level by applying a jet energy scale (JES) derived from simulation and *in situ* corrections based on 8 TeV data [58, 59]. Quality criteria are imposed to identify jets arising from non-collision sources or detector noise and any event containing such a jet is removed [60]. Further track-based selections are applied to reject jets with $p_{\text {T}} <60$ GeV and $|\eta |<2.4$ that originate from pile-up interactions [61] and the expected event average energy contribution from pile-up clusters is subtracted using a factor dependent on the jet area [58]. Jets are classified as “baseline” and “signal”. Baseline jets are required to have $p_{\text {T}} >20$ GeV and $|\eta |<2.8$. Signal jets, selected after resolving overlaps with electrons and muons, are required to pass the stricter requirement of $p_{\text {T}} >35$ GeV.

Jets are identified as $b$-jets if tagged by a multivariate algorithm which uses information about the impact parameters of inner detector tracks matched to the jet, the presence of displaced secondary vertices, and the reconstructed flight paths of *b*- and *c*-hadrons inside the jet [62]. The $b$-tagging working point with a 77 % efficiency, as determined in a simulated sample of $t\bar{t}$ events, was chosen as part of the optimization process discussed in Sect. 5. The corresponding rejection factors against jets originating from $c$-quarks and from light quarks and gluons at this working point are 4.5 and 130, respectively [63]. To compensate for differences between data and MC simulation in the $b$-tagging efficiencies and mis-tag rates, correction factors derived from data-driven methods are applied to the simulated samples [64]. Candidate *b*-tagged jets are required to have $p_{\text {T}} > 50$ GeV and $|\eta |<$ 2.5.

Electron candidates are reconstructed from energy clusters in the electromagnetic calorimeter matched to a track in the inner detector and are required to satisfy a set of “loose” quality criteria [65–67]. They are also required to lie within the fiducial volume $|\eta |<2.47$. Muon candidates are reconstructed from matching tracks in the inner detector and the muon spectrometer. Events containing one or more muon candidates that have a transverse (longitudinal) impact parameter with respect to the primary vertex larger than 0.2 mm (1 mm) are rejected to suppress cosmic rays. Muon candidates are also required to satisfy “medium” quality criteria [68] and have $|\eta |<$ 2.5. All electron and muon candidates must have $p_{\text {T}}>$ 10 GeV. Lepton candidates remaining after resolving the overlap with baseline jets (see next paragraph) are called “baseline” leptons. In the control regions where lepton identification is required, “signal” leptons are chosen from the baseline set with $p_{\text {T}}$ > 26 GeV to ensure full efficiency of the trigger and are required to be isolated from other activity using a selection designed to accept 99 % of leptons from *Z* boson decays. Signal electrons are further required to satisfy “tight” quality criteria [65]. Electrons (muons) are matched to the primary vertex by requiring the transverse impact parameter ($d_0$) to satisfy $|d_0/\sigma (d_0)|<$ 5 (3), and the longitudinal impact parameter ($z_0$) to satisfy $|z_0 \sin \theta |<$ 0.5 mm for both the electrons and muons. The MC events are corrected to account for differences in the lepton trigger, reconstruction and identification efficiencies between data and MC simulation.

The sequence to resolve overlapping objects begins by removing electron candidates sharing an inner detector track with a muon candidate. Next, jet candidates within $\Delta R=\sqrt{(\Delta y)^2 + (\Delta \phi )^2} =0.2$ of an electron candidate are discarded, unless the jet is $b$-tagged, in which case the electron is discarded since it is likely to originate from a semileptonic $b$-hadron decay. Finally, any lepton candidate remaining within $\Delta R = 0.4$ of any surviving jet candidate is discarded, except for the case where the lepton is a muon and the number of tracks associated with the jet is less than three.

The missing transverse momentum ${{\varvec{p}}}_{\mathrm {T}}^{\mathrm {miss}}$, with magnitude $E_{\mathrm {T}}^{\mathrm {miss}}$, is defined as the negative vector sum of the $p_{\text {T}}$ of all selected and calibrated physics objects in the event, with an extra term added to account for soft energy in the event which is not associated with any of the selected objects. This soft term is calculated from inner detector tracks with $p_{\text {T}}$ above 400 MeV matched to the primary vertex to make it more robust against pile-up contamination [69, 70].

Reconstructed photons, although not used in the main signal event selection, are selected in the regions employed in one of the alternative methods used to check the $$Z$$+jets background, as explained in Sect. 6. Photon candidates are required to have $$p_{\text {T}}$$  $$ > 130$$ GeV and $$|\eta | < 2.37$$, to satisfy the tight photon shower shape and electron rejection criteria [71], and to be isolated.

## Event selection

The selection of events is similar to that used in Ref. [72] and is based on the definition of one set of three overlapping signal regions (SRA) and a fourth distinct signal region (SRB). These were re-optimized for 3.2 fb$^{-1}$ of 13 TeV *pp* collisions, and the selection criteria are summarized in Table 1. Only events with $E_{\mathrm {T}}^{\mathrm {miss}}$ $>250$ GeV are retained to ensure full efficiency of the trigger. Jets are ordered according to decreasing $p_{\text {T}}$. Contamination from backgrounds with high jet multiplicity, particularly $t\bar{t}$ production, is suppressed by vetoing events with a fourth jet with $p_{\text {T}} >50$ GeV. To discriminate against multijet background, events where $E_{\mathrm {T}}^{\mathrm {miss}}$ is aligned with a jet in the transverse plane are rejected by requiring $\Delta \phi ^j_{\mathrm {min}}>0.4$, where $\Delta \phi ^j_{\mathrm {min}}$ is the minimum azimuthal distance between $E_{\mathrm {T}}^{\mathrm {miss}}$ and the leading four jets, and by requiring $E_{\mathrm {T}}^{\mathrm {miss}}/m_{\mathrm {eff}}>0.25$, where $m_{\mathrm {eff}}$ is defined as the scalar sum of the $E_{\mathrm {T}}^{\mathrm {miss}}$ and the $p_{\text {T}}$ of the two leading jets. For signal events, no isolated charged leptons are expected in the final state, and events where a baseline electron or muon is reconstructed are discarded.

**Table 1:** Summary of the event selection in each signal region. The term lepton is used in the table to refer to baseline electrons and muons. Jets ($j_1$, $j_2$, $j_3$ and $j_4$) are labelled with an index corresponding to their decreasing order in $p_{\text {T}}$

Variable	SRA	SRB
Event cleaning	Common to all SR	
Lepton veto	No *e*/$\mu$ with $p_{\text {T}}$ > 10 GeV after overlap removal	
$E_{\mathrm {T}}^{\mathrm {miss}}$ (GeV)	>250	>400
*n*(jets) $p_{\text {T}} > 35$ GeV	2–4	3–4
1st jet $p_{\text {T}} (j_1)$ (GeV)	>130	>300
2nd jet $p_{\text {T}} (j_2)$ (GeV)	>50	>50
4th jet	Vetoed if $p_{\text {T}} (j_4)>$ 50 GeV	
$\Delta \phi ^j_{\mathrm {min}}$	>0.4	>0.4
$\Delta \phi (j_1,E_{\mathrm {T}}^{\mathrm {miss}})$	–	>2.5
$b$-tagging	$j_1$ and $j_2$	$j_2$ and ($j_3$ or $j_4$)
$E_{\text {T}}^{\text {miss}}/m_{\mathrm {eff}}$	>0.25	>0.25
$m_\mathrm {CT}$ (GeV)	>250, 350, 450	–
$m_{bb}$ (GeV)	>200	–

The first set of signal regions, SRA, selects events with large $E_{\mathrm {T}}^{\mathrm {miss}}$ where the two leading jets are $b$-tagged to target models with a large mass difference between the bottom squark and the neutralino. The main discriminating variable is the contransverse mass ($m_\mathrm {CT}$) [73], which is a kinematic variable that can be used to measure the masses of pair-produced semi-invisibly decaying heavy particles. For two identical decays of heavy particles into two visible particles (or particle aggregates) $v_{1}$ and $v_{2}$, and two invisible particles, $m_\mathrm {CT}$ is defined as

$$\begin{aligned} m_\mathrm {CT} ^{2}(v_{1},v_{2}) = \left[ E_\mathrm{T}(v_{1}) + E_\mathrm{T}(v_{2}) \right] ^2 - \left[ {\mathbf{p }}_\mathrm{T}(v_{1}) - {\mathbf{p }}_\mathrm{T}(v_{2}) \right] ^2. \end{aligned}$$

In this analysis, $v_1$ and $v_2$ are the two leading $b$-jets. For signal events, these correspond to the $b$-jets from the squark decays and the invisible particles are the two neutralinos. The contransverse mass is invariant under equal and opposite boosts of the parent particles in the transverse plane. For systems of parent particles produced with small transverse boosts, $m_\mathrm {CT}$ is bounded from above by an analytical combination of particle masses. This bound is saturated when the two visible objects are collinear. For $t\bar{t}$ events, this kinematic bound is at 135 GeV, while for production of bottom squark pairs the bound is given by $m_\mathrm{CT}^\mathrm{max} = (m^2_{\tilde{b}^{}_{1} } - m^2_{\tilde{\chi }^{0}_{1}} ) / m_{\tilde{b}^{}_{1} }$. The selection on $m_\mathrm {CT}$ is optimized based on the bottom squark and neutralino masses considered and SRA is further divided into three overlapping regions, SRA250, SRA350 and SRA450, where the naming conventions reflects the minimum value allowed for $m_\mathrm {CT}$. Finally, a selection on the invariant mass of the two $b$-jets ($m_{bb}>200$ GeV) is applied to further enhance the signal yield over the SM background contributions. For a signal model corresponding to $m^{}_{\tilde{b}^{}_{1} }$ = 800 GeV and $m^{}_{\tilde{\chi }^{0}_{1}}$ = 1 GeV, 10, 8 and 5 % of the simulated signal events are retained by the SRA250, SRA350 and SRA450 selections, respectively.

The second type of signal region, SRB, selects events where a bottom squark pair is produced in association with a jet from initial-state radiation (ISR). The SRB targets models with a small mass difference between the $\tilde{b}^{}_{1}$ and the $\tilde{\chi }^{0}_{1}$, such that a boosted bottom squark pair is needed to satisfy the trigger requirements. Hence events are selected with large $E_{\mathrm {T}}^{\mathrm {miss}}$, one high-$p_{\text {T}}$ non-$b$-tagged leading jet and at least two additional $b$-jets. The leading jet is also required to be pointing in the direction opposite to the $E_{\mathrm {T}}^{\mathrm {miss}}$ by requiring $\Delta \phi (j_1,E_{\mathrm {T}}^{\mathrm {miss}}) > 2.5$, where $\Delta \phi (j_1,E_{\mathrm {T}}^{\mathrm {miss}})$ is defined as the azimuthal angle between the leading jet and the $E_{\mathrm {T}}^{\mathrm {miss}}$. For a signal model corresponding to $m^{}_{\tilde{b}^{}_{1} }$ = 400 GeV and $m^{}_{\tilde{\chi }^{0}_{1}}$ = 300 GeV, about 0.3 % of the simulated events are retained by the SRB selection.

**Table 2:** Definition of the control regions associated with SRA and SRB. Control regions are defined to study the contribution from $Z$+hf, $t\bar{t}$, single top-quark and $W$+hf production in SRA and the contribution from $Z$+hf and $t\bar{t}$ in SRB. The term lepton is used in the table to refer to signal electrons and muons. Jets ($j_1$, $j_2$, $j_3$ and $j_4$) are labelled with an index corresponding to their decreasing order in $p_{\text {T}}$. SFOS indicates the same-flavour opposite-sign two-lepton selection

Variable	CRzA	CRttA	CRstA	CRwA	CRzB	CRttB
Number of leptons	2 SFOS	1	1	1	2 SFOS	1
1st lepton $p_{\text {T}}$ (GeV)	>90	>26	>26	>26	>26	>26
2nd lepton $p_{\text {T}}$ (GeV)	>20	–	–	–	>20	–
$m_{\ell \ell }$ (GeV)	[76, 106]	–	–	–	[76, 106]	–
$m_{\mathrm {T}}$ (GeV)	–	–	–	>30	–	–
*n*(jets) $p_{\text {T}} > 35$ GeV	2–4	2–4	2–4	2–4	3–4	3–4
1st jet $p_{\text {T}} (j_1)$ (GeV)	>50	>130	>50	>130	>50	>130
2nd jet $p_{\text {T}} (j_2)$ (GeV)	>50	>50	>50	>50	>50	>50
4th jet	Vetoed if $p_{\text {T}} (j_4)> 50$ GeV				Vetoed if $p_{\text {T}} (j_4)> 50$ GeV	
$b$-tagged jets	$j_1$ and $j_2$	$j_1$ and $j_2$	$j_1$ and $j_2$	$j_1$	$j_2$ and ($j_3$ or $j_4$)	
$E_{\mathrm {T}}^{\mathrm {miss}}$ (GeV)	<100	>100	>100	>100	<70	>200
$E_{\mathrm {T}}^{\mathrm {miss,cor}}$ (GeV)	>100	–	–	–	>100	–
$m_{bb}$ (GeV)	>200	<200	>200	$(m_{bj}) > 200$	–	–
$m_\mathrm {CT}$ (GeV)	–	>150	>150	>150	–	–
$m_{b\ell }^{\mathrm {min}}$ (GeV)	–	–	>170	–	–	–
$\Delta \phi (j_1,~E_{\mathrm {T}}^{\mathrm {miss}})$	–	–	–	–	>2.0	>2.5

## Background estimation

The dominant SM background processes in the signal regions are the production of *W* or *Z* bosons in association with heavy-flavour jets (referred to as $W$+hf and $Z$+hf) and the production of top quarks. In particular, events with $Z$+hf production followed by the $Z\rightarrow \nu \bar{\nu }$ decay have the same signature as the signal and are the largest (irreducible) background in SRA. The background in SRB is dominated by top-quark production in events with a charged lepton in the final state that is not reconstructed, either because the lepton is a hadronically decaying $\tau$, or because the electron or muon is not identified or out of detector acceptance.

Monte Carlo simulation is used to estimate the background yield in the signal regions, after normalizing the Monte Carlo prediction for the major backgrounds to data in control regions (CR) constructed to enhance a particular background and to be kinematically similar but orthogonal to the signal regions. The control regions are defined by explicitly requiring the presence of one or two leptons (electrons or muons) in the final state and applying further selection criteria similar to those of the corresponding signal regions. To select events with good-quality electrons and muons the “signal lepton” selection is applied to them. Events with additional baseline lepton candidates are vetoed. For SRA, the normalizations of the top-quark pair, single-top-quark, $Z$+jets and $W$+jets backgrounds are estimated simultaneously by making use of four control regions, while for SRB two control regions are used to determine the normalization of the top-quark pair and $Z$+jets backgrounds since other contributions are sub-dominant. A likelihood function is built as the product of Poisson probability functions, using as constraints the observed and expected (from MC simulation) event yields in the control regions but not the yields in the corresponding SR [74]. The normalization factors for each of the simulated backgrounds are adjusted simultaneously via a profile likelihood fit [75] (referred to as the “background-only fit”). All systematic uncertainties (discussed in Sect. 7) are treated as nuisance parameters in the fit. The background normalization parameters are applied to the signal regions also taking into account correlations in the yield predictions between different regions.

Two same-flavour opposite-sign (SFOS) two-lepton (electron or muon) control regions with dilepton invariant mass near the *Z* boson mass ($76< m_{\ell \ell } <106$ GeV) and two $b$-tagged jets provide data samples dominated by *Z* boson production. For these control regions, labelled in the following as CRzA and CRzB for SRA and SRB respectively, the $p_{\text {T}}$ of the leptons is added vectorially to the ${{\varvec{p}}}_{\mathrm {T}}^{\mathrm {miss}}$ to mimic the expected missing transverse momentum spectrum of $Z\rightarrow \nu \bar{\nu }$ events, and is indicated in the following as $E_{\mathrm {T}}^{\mathrm {miss,cor}}$ (lepton corrected). In addition, the uncorrected $E_{\mathrm {T}}^{\mathrm {miss}}$ of the event is required to be less than 100 (70) GeV in CRzA (CRzB) in order to further enhance the *Z* boson contribution. In the case of CRzA, a $m_{bb}>200$ GeV selection is also imposed. Two control regions, CRttA and CRttB defined for SRA and SRB respectively, dominated by $t\bar{t}$ production are identified by selecting events with exactly one lepton ($e,\mu$) and a set of requirements similar to those for SRA and SRB, with the additional requirement $m_{bb}<200$ GeV in CRttA to separate the $t\bar{t}$ pair contribution from single top-quark production. To assist in the estimation of the background from $W$+hf and single top-quark production in SRA two further one-lepton control regions (CRwA and CRstA) are defined. These control regions exploit kinematic features to differentiate these processes from $t\bar{t}$ production. For CRstA a selection is applied to the minimum invariant mass of either of the $b$-jets and the charged lepton ($m_{b\ell }^{\mathrm {min}}$). For CRwA only one $b$-jet is required, the selection on $m_{bb}$ is replaced by a selection on the invariant mass of the two leading jets ($m_{bj}$) and a further selection is applied to the event transverse mass $m_{\mathrm {T}}$, defined as $m_{\mathrm {T}} = \sqrt{2 {p}_\mathrm {T}^\mathrm{lep} E_{\mathrm {T}}^{\mathrm {miss}}- 2 {{\varvec{p}}}_{\mathrm {T}}^{\mathrm {lep}} \cdot {{\varvec{p}}}_{\mathrm {T}}^{\mathrm {miss}}}$. The definitions of the control regions are summarized in Table 2.

The contributions from diboson and $t\bar{t} + W/Z$ processes are minor and they are collectively called “Others” in the following. They are estimated from MC simulation for both the signal and the control regions and included in the fit procedure, and are allowed to vary within their uncertainty. The background from multijet production is estimated from data using a procedure described in detail in Ref. [76] and modified to account for the flavour of the jets. The procedure consists of smearing the jet response in events with well-measured $E_{\mathrm {T}}^{\mathrm {miss}}$ (seed events). The jet response function is obtained from MC dijet events and cross-checked in events from data where the $E_{\mathrm {T}}^{\mathrm {miss}}$ can be unambiguously attributed to the mis-measurement of one of the jets. The contribution from multijet production in all regions is found to be negligible.

**Table 3:** Fit results in the control regions associated with the SRA and SRB selection for an integrated luminosity of 3.2 fb$^{-1}$. The results are obtained from the control regions using the background-only fit. The uncertainties include statistical, detector-related and theoretical systematic components. The individual uncertainties can be correlated and do not necessarily add in quadrature to the total systematic uncertainty. The pure MC estimate is used for backgrounds for which a dedicated CR is not defined, e.g. for smaller backgrounds (indicated as “Other”) and for single top-quark and $W$+jets production in CRzB and CRttB. A dash indicates a negligible background

Control region	CRzA	CRwA	CRttA	CRstA	CRzB	CRttB
Observed	78	543	260	56	59	188
Total background (fit)	${78} \pm {9}$	${543} \pm {23}$	${260} \pm {16}$	${56} \pm {7}$	${59} \pm {8}$	${188} \pm {14}$
$t\bar{t}$	$9.0 \pm 1.6$	${153} \pm {26}$	${181} \pm {23}$	${11.1} \pm {2.1}$	${14.6} \pm {2.0}$	${156} \pm {15}$
Single top	$0.8 \pm 0.4$	$50 \pm 23$	$27 \pm 13$	$23 \pm 10$	$0.42 \pm 0.07$	${16.6} \pm {2.0}$
*W*+jets	–	${327} \pm {43}$	$45 \pm 14$	${20} \pm {6}$	–	${13} \pm {5}$
*Z*+jets	${68} \pm {9}$	$3.8 \pm 0.6$	$1.4 \pm 0.2$	$0.9 \pm 0.2$	${42} \pm {8}$	$0.3 \pm 0.1$
“Other”	$0.9 \pm 0.1$	$8.1 \pm 1.1$	$5.8 \pm 0.7$	$0.6 \pm 0.1$	$1.6 \pm 0.4$	$2.3 \pm 0.2$
Total background (MC exp.)	61	503	267	57	46	191
$t\bar{t}$	9.4	161	190	12	15	159
Single top	1.1	60	33	27	0.4	16
*W*+jets	–	270	37	17	–	12.9
*Z*+jets	50	2.8	1.0	0.7	29	0.2
“Other”	0.9	8.1	5.8	0.6	1.6	2.3

**Fig. 1:**
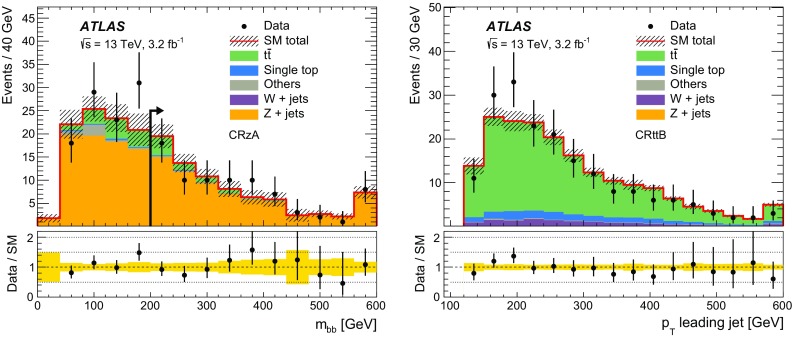
*Left* $m_{bb}$ distribution in CRzA before the final selection $m_{bb}>200$ GeV is applied (indicated by the *arrow*). *Right* $p_{\text {T}}$ of the leading jet in CRttB. In both distributions the MC normalization is rescaled using the results from the background-only fit, showing good agreement between data and the predicted SM shapes. The *shaded band* includes statistical and detector-related systematic uncertainties as detailed in Sect. 7 and the last bin includes overflows

The results of the background-only fit are shown in Table 3, where the contribution from individual backgrounds is shown separately as a purely MC-based prediction and with the rescaling from the fit procedure. The $m_{bb}$ distribution in CRzA and the $p_{\text {T}}$ of the leading jet in CRttB are shown in Fig. 1 after the backgrounds were rescaled as a result of the background-only fit, showing good agreement in the shape of the distributions in the two control regions used to estimate the dominant backgrounds in SRA and SRB.

The full background estimation procedure is validated by comparing the background predictions and the shapes of the distributions of the key analysis variables from the fit results to those observed in dedicated validation regions. They are defined to be mutually exclusive and kinematically similar to the signal regions, with low potential contamination from signal. For SRA, two validation regions are defined by using the same definition as SRA250, and inverting the selections on $m_{bb}$ and $m_\mathrm {CT}$. The SM predictions are found to overestimate the data by about one standard deviation. For SRB, a validation region is defined by selecting events with 250 $<E_{\mathrm {T}}^{\mathrm {miss}}<$ 300 GeV and good agreement is observed between data and predictions.

As a further validation, two alternative methods are used to estimate the $Z$+hf contribution. The first method exploits the similarity of the $Z$+jets and $\gamma +$jets processes [76]. For $p_{\text {T}}$ of the photon significantly larger than the mass of the *Z* boson, the kinematics of $\gamma +$jets events strongly resemble those of $Z$+jets events. The event yields are measured in control regions identical to the SRA and SRB, with the $E_{\mathrm {T}}^{\mathrm {miss}}$-based selections replaced by selections on the $p_{\text {T}}$ of the photon vectorially added to the ${{\varvec{p}}}_{\mathrm {T}}^{\mathrm {miss}}$. The yields are then propagated to the actual SRA and SRB using a reweighting factor derived using the MC simulation. This factor takes into account the different kinematics of the two processes and residual effects arising from the acceptance and reconstruction efficiency for photons.

In the second alternative method, applied to SRA only where the $Z$+hf contribution is dominant, the MC simulation is used to verify that the shape of the $m_\mathrm {CT}$ distribution for events with no $b$-tagged jets is compatible with the shape of the $m_\mathrm {CT}$ distribution for events where two *b*-tagged jets are present. A new highly populated $Z$+jets CR is defined selecting $Z\rightarrow \ell \ell$ events with no *b*-tagged jets. The $m_\mathrm {CT}$ distribution in this CR is constructed using the two leading jets and is used to estimate the shape of the $m_\mathrm {CT}$ distribution in the SRA, whilst the normalization in SRA is rescaled based on the ratio in data of $Z \rightarrow \ell \ell$ events with no $b$-tagged jets to events with two $b$-tagged jets. Additional MC-based corrections are applied to take into account the two-lepton selection in this CR.

**Table 4:** Estimated $Z$+jets yields in the signal regions as obtained using the default and the two alternative methods. The errors include all the uncertainty sources discussed in Sect. 7. The “MC-based post-fit” uncertainty does not include the additional uncertainty to account for the difference between the three methods

Method\region	SRA250	SRA350	SRA450	SRB
Nominal MC-based post-fit	$22 \pm 4$	$5.0 \pm 0.9$	$1.3 \pm 0.3$	$4.1 \pm 0.9$
*Z*+jets from $\gamma +$jets events	$18 \pm 5$	$3.7 \pm 1.5$	$1.8 \pm 1.0$	$2.2 \pm 1.0$
*Z*+jets from non *b*-tagged *Z* events	$18 \pm 6$	$4.3 \pm 1.6$	$1.3 \pm 0.5$	Not applicable

The two alternative methods are in agreement within uncertainties with the estimates obtained with the profile likelihood fit to the control regions (Table 4). Experimental and theoretical systematic uncertainties in the nominal and alternative method estimates are taken into account (see Sect. 7). The difference between the alternative methods and the background-only fit is taken into account as an additional systematic uncertainty in the final $Z$+hf yields.

## Systematic uncertainties

Several sources of experimental and theoretical systematic uncertainty are considered in this analysis. Their impact is reduced through the normalization of the dominant backgrounds in the control regions with kinematic selections resembling those of the corresponding signal region (see Sect. 6). Uncertainties due to the limited number of events in the CRs are also taken into account in the fit. The individual contributions are outlined in Table 5.

**Table 5:** Summary of the dominant experimental and theoretical uncertainties for each signal region. Uncertainties are quoted as relative to the total uncertainty, with a range indicated for the three SRAs. For theoretical modelling, uncertainties per dominant SM background process are quoted. The individual uncertainties can be correlated, and do not necessarily add in quadrature to the total background uncertainty

Source\region	SRA (%)	SRB (%)
Experimental uncertainty		
JES	15–30	25
JER	20–35	10
*b*-tagging	25–45	15
Theoretical modelling uncertainty		
*Z*+jets	25–35	12
*W*+jets	20–22	27
Top production	15–20	70

The dominant detector-related systematic effects are due to the uncertainties in the jet energy scale (JES) [58] and resolution (JER) [59], and in the *b*-tagging efficiency and mis-tagging rates. The JES and JER uncertainties are estimated from 13 TeV data, while the uncertainties related to $b$-tagging are estimated from 8 TeV data and extrapolated to 13 TeV and to the Run-2 detector conditions. The uncertainties associated with lepton and photon reconstruction and energy measurements are also considered but have a small impact on the final results. Lepton, photon and jet-related uncertainties are propagated to the calculation of the $E_{\mathrm {T}}^{\mathrm {miss}}$, and additional uncertainties are included in the energy scale and resolution of the soft term. The overall experimental uncertainty in the SM background estimate is found to be around 20 % for the SRAs and 15 % for the SRB.

Uncertainties in the modelling of the SM background processes from MC simulation and their theoretical cross-section uncertainties are also taken into account. The dominant uncertainty arises from *Z*+jets MC modelling for SRA and $t\bar{t}$ modelling for SRB. The *Z*+jets (as well as *W*+jets) modelling uncertainties are evaluated using alternative samples generated with different renormalization and factorization scales, merging (CKKW-L) and resummation scales. An additional one-sided uncertainty in the $Z$+jets estimate is taken as the largest deviation between the nominal background-only fit result and each of the alternative data-driven estimates described in Sect. 6. This results in an additional 25, 25 and 40 % one-sided uncertainty in SRA250, SRA350 and SRA450, respectively. Finally, a 40 % uncertainty [77] is assigned to the heavy-flavour jet content in *W*+jets, estimated from MC simulation in SRB. For SRA, the uncertainty accounts for the different requirements on $b$-jets between CRwA and the signal region.

**Table 6:** Fit results in all signal regions for an integrated luminosity of 3.2 fb$^{-1}$. The results are obtained from the control regions. The background normalization parameters obtained with the background-only fit are applied to the SRs. The individual uncertainties, including detector-related and theoretical systematic components, are symmetrized and can be correlated and do not necessarily add in quadrature to the total systematic uncertainty

Signal region	SRA250	SRA350	SRA450	SRB
Observed	23	6	1	6
Total background (fit)	${29} \pm {5}$	$7.0 \pm 1.2$	$1.8\pm 0.4$	${12.0} \pm {2.5}$
$t\bar{t}$	${1.0} \pm {0.4}$	$0.17 \pm 0.08$	$0.04 \pm 0.02$	$5.5 \pm 2.0$
Single top	$1.8 \pm 1.0$	$0.53 \pm 0.30$	$0.13 \pm 0.07$	$1.0 \pm 0.4$
*W*+jets	$4.4 \pm 1.3$	$1.2 \pm 0.4$	$0.30 \pm 0.10$	$1.1 \pm 0.6$
*Z*+jets	$\text{[ }2ex]{22} \pm \text{[ }2ex]{4}$	$5.0 \pm 1.1$	$1.3 \pm 0.4$	$4.1 \pm 1.3$
“Other”	$0.45 \pm 0.06$	$0.14 \pm 0.04$	$0.04 \pm 0.04$	$0.3 \pm 0.1$
Total background (MC exp.)	23	5.6	1.5	11
$t\bar{t}$	1.1	0.18	0.04	5.6
Single top	2.2	0.6	0.15	1.0
*W*+jets	3.6	1.0	0.25	1.1
*Z*+jets	16	3.7	1.0	2.8
“Other”	0.45	0.14	0.04	0.3

Uncertainties in the modelling of the top-quark pair and single-top-quark (*Wt*) backgrounds are sub-dominant in SRA and dominant in SRB. They are computed as the difference between the predictions from nominal samples and those of additional samples differing in generator or parameter settings. Hadronization and PS uncertainties are estimated using samples generated with Powheg-Box v2 and showered by Herwig++ v2.7.1 [78] with the UEEE5 underlying event tune. Uncertainties related to initial- and final-state radiation modelling, tune and (for $t\bar{t}$  only) choice of $h^{}_\mathrm{damp}$ parameter in Powheg-Box v2 are evaluated using alternative settings of the generators. Finally, an alternative generator MadGraph5_aMC@NLO with showering by Herwig++ v2.7.1 is used to estimate the generator uncertainties. Uncertainties in smaller backgrounds such as diboson and *ttV* are also estimated by comparisons of the nominal sample with alternative samples differing in generator or parameter settings (Powheg v2 with showering by Pythia  8.210 for diboson, renormalization and factorization scale and A14 tune variations for *ttV*) and are found to be negligible. The cross-sections used to normalize the MC yields to the highest order available are varied according to the scale uncertainty of the theoretical calculation, i.e. 5 % for *W*, *Z* boson and top-quark pair production, 6 % for diboson, 13 and 12 % for *ttW* and *ttZ*, respectively.

For the SUSY signal processes, both the experimental and theoretical uncertainties in the expected signal yield are considered. Experimental uncertainties, which are found to be between 20 and 25 % across the $\tilde{b}^{}_{1}$–$\tilde{\chi }^{0}_{1}$ mass plane for all SRs, are largely dominated by the uncertainty in the *b*-tagging efficiency in SRA, while JER and *b*-tagging uncertainties are dominant in SRB with equal contributions. Theoretical uncertainties in the NLO + NLL cross-section are calculated for each SUSY signal scenario and are dominated by the uncertainties in the renormalization and factorization scales, followed by the uncertainty in the PDF. They vary between 15 and 20 % for bottom squark masses in the range between 400 and 900 GeV. Additional uncertainties in the modelling of initial-state radiation in SUSY signal MC samples are taken into account and contribute up to 5 %.

## Results and interpretation

**Fig. 2:**
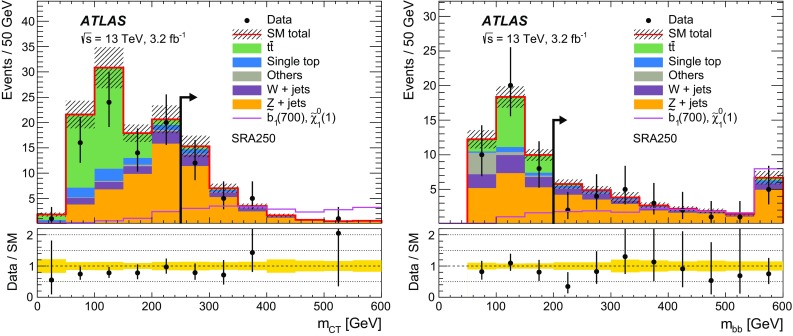
*Left* $m_\mathrm {CT}$ distribution in SRA250 with all the selection criteria applied except the $m_\mathrm {CT}$ threshold. *Right* $m_{bb}$  distribution in SRA250 with all selection criteria applied except the $m_{bb}$ requirement. The *arrows* indicate the final applied selection. The *shaded band* includes statistical and detector-related systematic uncertainties. The SM backgrounds are normalized to the values determined in the fit. For illustration the distributions expected for one signal model with *bottom squark* and neutralino masses of 700 and 1 GeV, respectively, are overlaid. The last bin includes overflows

**Fig. 3:**
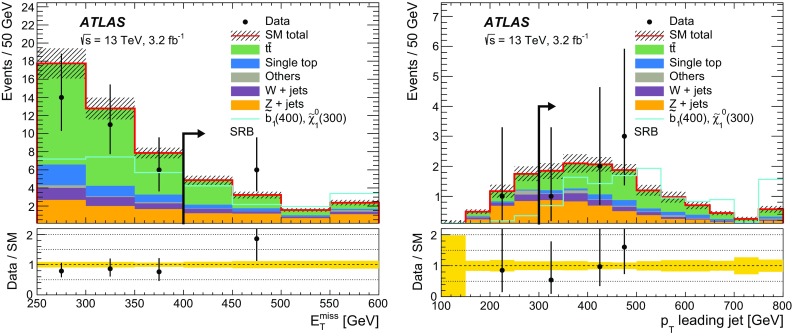
*Left* $E_{\mathrm {T}}^{\mathrm {miss}}$ distribution in SRB with all the selection criteria applied except the $E_{\mathrm {T}}^{\mathrm {miss}}$ threshold. *Right* leading jet $p_{\text {T}}$ distribution in SRB with all the selection criteria applied except the selection on the leading jet $p_{\text {T}}$ itself. The *arrows* indicate the final selection applied in SRB. The *shaded band* includes statistical and detector-related systematic uncertainties. The SM backgrounds are normalized to the values determined in the fit. For illustration the distributions expected for one signal model with *bottom squark* and neutralino masses of 400 and 300 GeV, respectively, are overlaid. The last bin includes overflows

Table 6 reports the observed number of events and the SM predictions after the background-only fit for each signal region. The largest background contribution in SRA arises from $Z\rightarrow \nu \bar{\nu }$ produced in association with *b*-quarks whilst top-quark pair production dominates SM predictions for SRB. The background-only fit results are compared to the pre-fit predictions based on MC simulation. Figures 2 and 3 show the comparison between the observed data and the SM predictions for some relevant kinematic distributions in SRA250 and SRB, respectively, prior to the selection on the variable shown. For illustrative purposes, the distributions expected for a scenario with bottom squark and neutralino masses of 700 GeV (400 GeV) and 1 GeV (300 GeV), respectively, are shown for SRA250 (SRB). No excess above the expected Standard Model background yield is observed. The results are translated into upper limits on contributions from new physics beyond the SM (BSM) for each signal region. The CL$_s$ method [79, 80] is used to derive the confidence level of the exclusion; signal models with a CL$_s$ value below 0.05 are said to be excluded at 95 % CL. The profile-likelihood-ratio test statistic is used to exclude the signal-plus-background hypothesis for specific signal models. When normalized by the integrated luminosity of the data sample, results can be interpreted as corresponding upper limits on the visible cross-section, $\sigma ^{}_\mathrm{vis}$, defined as the product of the BSM production cross-section, the acceptance and the selection efficiency of a BSM signal. Table 7 summarizes the observed ($S^{95}_\mathrm {obs}$) and expected ($S^{95}_\mathrm {exp}$) 95 % CL upper limits on the number of BSM events and on $\sigma ^{}_\mathrm{vis}$.

**Fig. 4:**
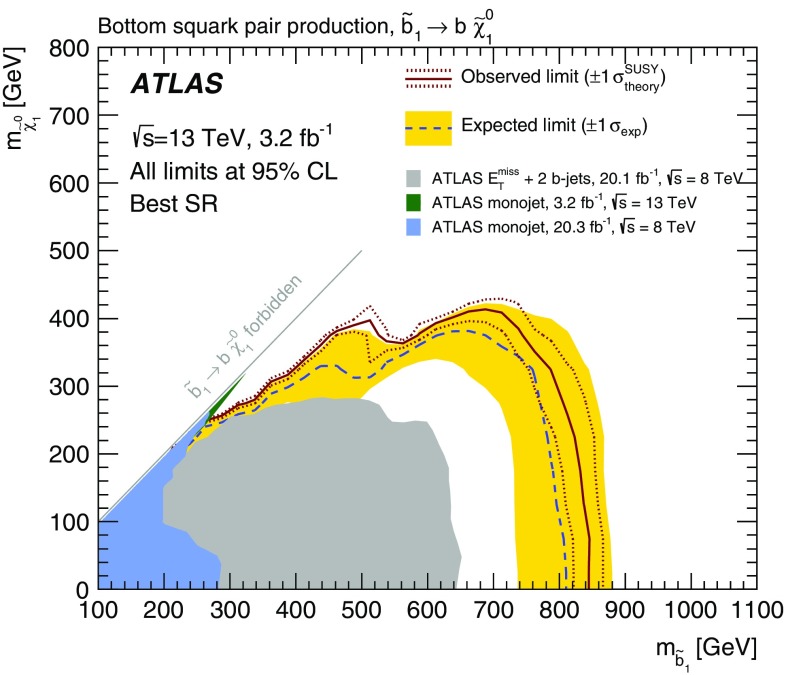
Observed and expected exclusion limits at 95 % CL, as well as $\pm 1 \sigma$ variation of the expected limit, in the $\tilde{b}^{}_{1}$–$\tilde{\chi }^{0}_{1}$ mass plane. The SR with the best expected sensitivity is adopted for each point of the parameter space. The *yellow band* around the expected limit (*dashed line*) shows the impact of the experimental and SM background theoretical uncertainties. The *dotted lines* show the impact on the observed limit of the variation of the nominal signal cross-section by $\pm 1 \sigma$ of its theoretical uncertainties. The exclusion limits from the Run-1 ATLAS searches [72, 81] and from the 13 TeV monojet search [82] are also superimposed. The latter limit is only published for values of $m_{\tilde{b}^{}_{1} }- m_{\tilde{\chi }^{0}_{1}}=$ 5 and 20 GeV

Exclusion limits are obtained assuming a specific SUSY particle mass hierarchy such that the lightest bottom squark decays exclusively via $\tilde{b}^{}_{1} \rightarrow b \tilde{\chi }^{0}_{1}$. In this case, the fit procedure takes into account not only correlations in the yield predictions between control and signal regions due to common background normalization parameters and systematic uncertainties but also contamination in the control regions from SUSY signal events (found to be generally negligible). The experimental systematic uncertainties in the signal are taken into account for this calculation and are assumed to be fully correlated with those in the SM background. Figure 4 shows the observed (solid line) and expected (dashed line) exclusion contours at 95 % CL in the $\tilde{b}^{}_{1}$–$\tilde{\chi }^{0}_{1}$ mass plane.

**Table 7:** Left to right: 95 % CL upper limits on the visible cross-section ($\langle \epsilon A \sigma \rangle _\mathrm{obs}^{95}$) and on the number of signal events ($S_\mathrm{obs}^{95}$ ). The third column ($S_\mathrm{exp}^{95}$) shows the 95 % CL upper limit on the number of signal events, given the expected number (and $\pm 1\sigma$ variations of the expectation) of background events

Signal channel	$\langle \epsilon A \mathrm{\sigma }\rangle _\mathrm{obs}^{95}$ (fb)	$S_\mathrm{obs}^{95}$	$S_\mathrm{exp}^{95}$
SRA250	3.42	11.0	${13.8}^{+6.0}_{-3.2}$
SRA350	1.93	6.2	${6.6}^{+3.1}_{-1.1}$
SRA450	1.23	3.9	${4.1}^{+1.9}_{-0.6}$
SRB	1.89	6.1	${8.7}^{+3.1}_{-2.5}$

At each point of the parameter space, the SR with the best expected sensitivity is adopted. Sensitivity to scenarios with the largest mass difference between the $\tilde{b}^{}_{1}$ and the $\tilde{\chi }^{0}_{1}$ is achieved with the most stringent $m_\mathrm {CT}$  threshold (SRA450). Sensitivity to scenarios with smaller mass differences is achieved predominantly with searches based on the presence of a high-$p_{\text {T}}$ ISR jet, as in the dedicated SRB selection and the search described in Ref. [81]. Bottom squark masses up to 800 (840) GeV are excluded for $\tilde{\chi }^{0}_{1}$ masses below 360 (100) GeV. Differences in mass above 100 GeV between $\tilde{b}^{}_{1}$ and $\tilde{\chi }^{0}_{1}$ are excluded up to $\tilde{b}^{}_{1}$ masses of 500 GeV. The expected exclusion constraints are about 20 GeV lower than the observation for high bottom squark masses and about 60 GeV lower for SUSY models with small $\tilde{b}^{}_{1}$–$\tilde{\chi }^{0}_{1}$ mass splitting. The current results significantly extend the $\sqrt{s} = 8$ TeV limits [72, 81] despite the lower integrated luminosity mostly because of the increase in centre-of-mass energy of the LHC. Furthermore, the sensitivity of the analysis benefits from the advanced algorithms adopted to identify *b*-jets and use of information from the newly installed IBL pixel layer in the Run-2 ATLAS detector, as well as from improved techniques to estimate SM background contributions and their systematic uncertainties.

## Conclusion

In summary, the results of a search for bottom squark pair production are reported. The analysis uses 3.2 fb$^{-1}$  of *pp* collisions at $\sqrt{s}=13$ TeV collected by the ATLAS experiment at the Large Hadron Collider in 2015. Bottom squarks are searched for in events containing large missing transverse momentum and up to four jets, exactly two of which are identified as $b$-jets. No excess above the expected Standard Model background yield is found. Exclusion limits at 95 % confidence level are placed on the visible cross-section and on the mass of the bottom squark in phenomenological supersymmetric *R*-parity-conserving models in which the $\tilde{b}^{}_{1}$ is the lightest squark and is assumed to decay exclusively via $\tilde{b}^{}_{1} \rightarrow b \tilde{\chi }^{0}_{1}$, where $\tilde{\chi }^{0}_{1}$ is the lightest neutralino. Bottom squark masses up to 800 GeV are excluded for $\tilde{\chi }^{0}_{1}$ masses below 360 GeV (840 GeV for $\tilde{\chi }^{0}_{1}$ masses below 100 GeV) whilst differences in mass above 100 GeV between $\tilde{b}^{}_{1}$ and $\tilde{\chi }^{0}_{1}$ are excluded up to $\tilde{b}^{}_{1}$ masses of 500 GeV. The results significantly extend the constraints on bottom squark masses with respect to Run-1 searches.

## Footnotes

1 Scalar partners of the left-handed and right-handed chiral components of the bottom quark ($\tilde{b}_{\mathrm {L},\mathrm {R}}$) or top quark ($\tilde{t}_{\mathrm {L},\mathrm {R}}$) mix to form mass eigenstates for which $\tilde{b}^{}_{1}$ and $\tilde{t}^{}_{1}$are defined as the lighter of the two.

2 ATLAS uses a right-handed coordinate system with its origin at the nominal interaction point in the centre of the detector. The positive *x*-axis is defined by the direction from the interaction point to the centre of the LHC ring, with the positive *y*-axis pointing upwards, while the beam direction defines the *z*-axis. Cylindrical coordinates $(r,\phi )$ are used in the transverse plane, $\phi$ being the azimuthal angle around the *z*-axis. The pseudorapidity $\eta$ is defined in terms of the polar angle $\theta$ by $\eta =-\ln \tan (\theta /2)$. Rapidity is defined as *y* = $0.5 \ln [(E + p_z)/(E-p_z)]$ where *E* denotes the energy and $p_z$ is the component of the momentum along the beam direction.
